# Optimization of Cas9 RNA sequence to reduce its unexpected effects as a microRNA sponge

**DOI:** 10.1186/s12943-022-01604-x

**Published:** 2022-06-24

**Authors:** Junfeng Jiang, Tao Zeng, Li Zhang, Xingfei Fan, Qishu Jin, Haitao Ni, Yusheng Ye, Lipeng Cheng, Li Li, Liujun Wang, Sha Xu, Yu Yang, Juan Gu, Bing Guo, Lei Wang, Xin Li, Yingyi Qin, Jiaxi Li, Jinjiang Wang, Xi Chen, Minjuan Wu, Qi-long Ying, Xingjun Qin, Yefei Wang, Yue Wang

**Affiliations:** 1 Histology and Embryology Department, Naval Medical University, Shanghai, 200433 China; 2 Research Center of Developmental Biology, Shanghai Key Laboratory of Cell Engineering, Naval Medical University, 800 Xiangyin Road, Shanghai, 200433 China; 3 The 901th Hospital of PLA Jiont Logistic Support Force, HeFei, 230031 China; 4 Department of Pathology, Faculty of Medical Imaging, Naval Medical University, Shanghai, 200433 China; 5 Department of Histology and Embryology, Harbin Medical University, Harbin, 150086 China; 6 Department of Oral & Maxillofacial - Head & Neck Oncology, Shanghai Ninth People’s Hospital, College of Stomatology, Shanghai Jiao Tong University School of Medicine, Shanghai, 200011 China; 7 National Clinical Research Center for Oral Diseases, Shanghai, 200011 China; 8 Shanghai Ninth People’s Hospital, Shanghai Jiao Tong University School of Medicine, Shanghai Key Laboratory of Stomatology, 639 Zhi Zao Ju Road, Shanghai, 200011 China; 9 Department of health statistics, Naval Medical University, Shanghai, 200433 China; 10 Department of Oncology, Tongren Hospital, Shanghai Jiao Tong University School of Medicine, Shanghai, 200336 China; 11 Department of Cell and Neurobiology, Eli and Edythe Broad Center for Regenerative Medicine and Stem Cell Research at USC, Keck School of Medicine, University of Southern California, Los Angeles, California 90033 USA; 12 Department of Ophthalmology, Ninth People’s Hospital, Shanghai Jiao Tong University School of Medicine, Shanghai, 200011 China; 13 Shanghai Key Laboratory of Orbital Diseases and Ocular Oncology, Shanghai, 200011 China

**Keywords:** CRISPR–Cas9, miRNA sponge, Let-7, RNA sequence optimization

## Abstract

Cas9 RNA functions as a miRNA sponge.

Let-7 is the dominant regulated miRNA by Cas9 RNA.

RNA sequence optimization of Cas9 by synonymous mutation improves its safety.

**Supplementary Information:**

The online version contains supplementary material available at 10.1186/s12943-022-01604-x.

## Main text

CRISPR–Cas9 gene-editing technology has a wide range of potential future applications, including cancer treatment [1]. Currently, the main safety concerns about CRISPR–Cas9 technology are the host immune response to its components [2, 3] and off-target modifications [4]. However, safety concerns at the RNA level have not been evaluated.

A wide range of interactions between microRNAs (miRNAs) and mRNAs occur, not only in the 3′ untranslated regions but also in the amino acid coding sequences (CDSs) of mRNA [5, 6]. An increasing number of research groups [7, 8], including our team [9–11], recently found that a long RNA can bind and adsorb partially complementary miRNAs through a “miRNA sponge” mechanism, upregulating other target genes inhibited by these miRNAs. In this study, we investigated whether Cas9 RNA, an exogenous long-chain nucleic acid substance, could affect host cell gene expression through a miRNA sponge mechanism and developed an RNA sequence optimization strategy to improve the safety of this gene-editing system.

## Cas9 RNA could affect intracellular gene expression through a miRNA sponge mechanism

**Fig. 1:**
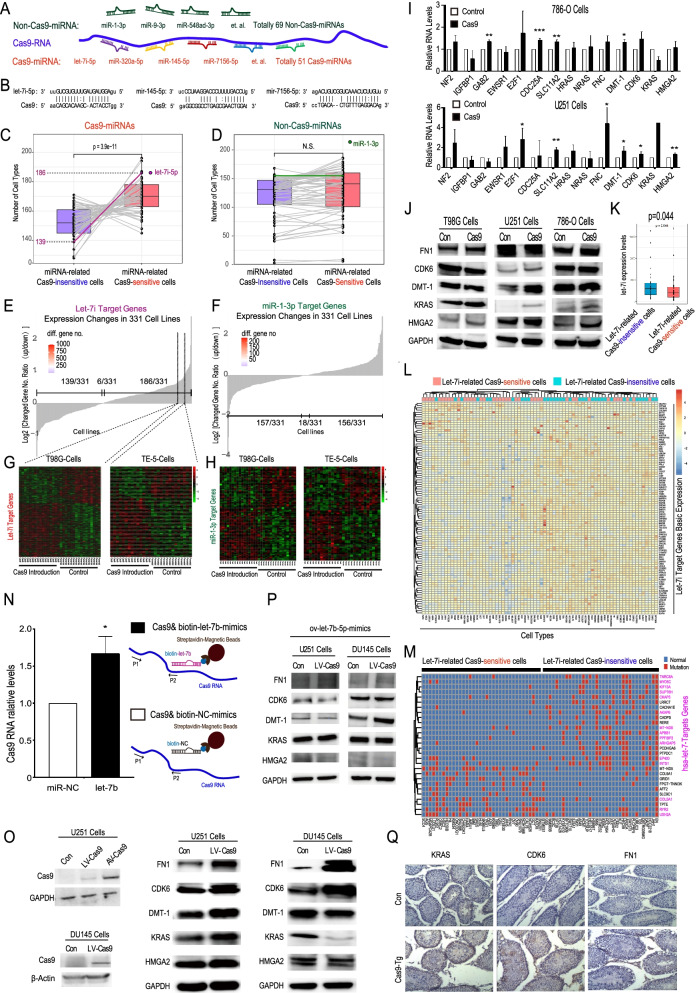
Cas9 RNA showed a trend of disturbing endogenous genes through a miRNA sponge mechanism. **A** Pattern diagram showing the binding relationship between Cas9 RNA and representative miRNAs from the Cas9-miRNA group or non-Cas9-miRNA group. The Cas9 RNA sequence is based on pLX311-Cas9 [12], Addgene #118018. The 51 Cas9-miRNAs were those that could be predicted to bind Cas9 RNA under strict standards in miRanda software: the value of the score parameter was higher than 160, and the value of the energy parameter was lower than − 25 kCal/Mol. The 69 non-Cas9-miRNAs were those that could not be predicted to bind Cas9 RNA under loose standards: the value of the score parameter was lower than 60, and the value of the energy parameter was higher than − 5 kCal/Mol. **B** Binding sites of representative Cas9-miRNAs and Cas9 RNA. **C**-**D** Overall features of 51 Cas9-miRNAs (**C**) or 69 non-Cas9-miRNAs (**D**) target genes expression changes due to the introduction of Cas9 in 331 cell lines. The cells sensitive to the Cas9-induced miRNA sponge effect were identified by the expression profiles of a certain miRNA’s target genes, which should be in an overall up-regulated trend after Cas9 introduction. The Cas9-insensitive cells were also identified in the opposite way. For each miRNA, the numbers of its sensitive or insensitive cell lines were compared basing on a transcription dataset of 331 cell lines before and after Cas9 introduction. For an example of Cas9-miRNAs, the target genes of let-7i (highlighted in purple) showed up-regulated trends in 186 types of cells after Cas9 introduction while in only 139 cell types with down-regulated trends. MiR-1-3p (highlighted in green) is an example of non-Cas9-miRNAs. *P* < 0.01 was considered to indicate a statistically significant. N.S., no statistical significance. **E** Overall view of the let-7i target genes expression profiles in 331 cell lines. Each bar represents the profile in one cell type, and the value of the bar is the log2 value of the ratio of upregulated gene number to downregulated gene number. For example, in T98G cells, 290 let-7i target genes were upregulated (*P* < 0.05, t test), and 194 let-7i target genes were downregulated (*P* < 0.05, t test) after Cas9 introduction; thus, the bar value is log2(290/194) =0.58. **F** Overall view of the miR-1-3p target genes expression profiles in 331 cell lines. **G**-**H** Heatmaps of the expression levels of let-7i target genes (**G**) or miR-1-3p target genes (**H**) before and after Cas9 introduction into two example cell types: T98G cells and TE-5 cells. **I** RT–PCR analysis of the RNA expression level changes of representative reported let-7 target genes after Cas9 introduction in 786-O and U251 cells. The data are presented as the mean ± SD. *, *p* < 0.05, **, *p* < 0.01, ***, *p* < 0.001, t test, *n* = 3. **J** Western blot analysis of the protein expression level changes of representative reported let-7 target genes after Cas9 introduction into T98G, U251 and 786-O cells. Cas9 was introduced into cells by adding lentivirus expressing the same Cas9 RNA that was introduced into the 331 cell lines. **K**-**L** Visual display of the let-7i expression levels (**K**) and heatmap of the differently expressed target genes of let-7i (**L**) in two cell groups. Let-7i-related Cas9 sensitive cells Group: the top 35 types of cells in which more let-7i target genes were increased after Cas9 introduction. Let-7i-related Cas9 insensitive cells Group: the top 35 cell types in which let-7i target genes were not increased after Cas9 introduction. It is shown that the basic expression of these let-7i target genes are relatively low in the Sensitive Group cells (**L**). **M** Heatmap showed the distribution of the 28 high-frequency mutant genes (mutation frequency > 10%) in the let-7i-related Cas9 sensitive and insensitive group. The reported let-7 target genes were marked purple. Almost all high-frequency mutant let-7 target genes were distributed in the “Cas9-insensitive” cells. **N** RNA pull-down strategy and results in HEK293 cells. Cas9-expressing lentivirus-infected HEK293 cells were transfected with biotin-labeled-miR-let-7-mimic or biotin-labeled-miR-NC-mimic. RNA was pulled down using Streptavidin Magnetic Beads and then subjected to qPCR analysis. The data are presented as the mean ± SD. *, *p* < 0.05, t test, *n* = 3. **O** Western blot analysis of the protein expression level changes of Cas9 and representative reported let-7 target genes in U251 and DU145 cells after the introduction of the Cas9 used in our laboratory. AV: adenovirus, LV: lentivirus. **P** Western blot analysis of some let-7 target genes in U251 and DU145 cells after introduction of Cas9 with let-7-mimic. **Q** Immunohistochemical analysis of KRAS, CDK6 and FN1 protein levels in the testes of Cas9-transgenic mice and control mice. Cas9-Tg: Cas9-transgenic mouse

To analyze the regulatory effect of Cas9 on its predicted binding microRNAs and their target genes, we first mined a database with the transcriptome characteristics of 331 cell lines overexpressing a Cas9 RNA and their parental control counterparts [12]. We also analyzed the binding possibilities between this Cas9 RNA and all the known human miRNAs using miRanda software [8]. The 51 miRNAs that were predicted to have the highest likelihood of binding Cas9 RNA were named “Cas9-miRNAs” (Fig. 1A-B and Table S1), while the 69 miRNAs predicted not to bind Cas9 were named “non-Cas9-miRNAs” (Fig. 1A and Table S2). According to the microRNA sponge hypothesis, the expression levels of a certain miRNA’s targeting genes should be increased in cells into which Cas9 is introduced, and these cells could be identified as this miRNA related Cas9-sensitive cells, and vice versa. For most Cas9-miRNAs in most cell types, the change trends of their target genes were consistent with this hypothesis (Fig. 1C, Fig. S1). For example, more of the target genes of let-7i were upregulated after Cas9 introduction in 186 cell types, which should be identified as let-7i-related Cas9-sensitive cells, such as the T98G, TE-5 and 786-O cell lines (Fig. 1E, G, Fig. S1). Noteworthy, for the overall feature of 51 Cas9-miRNAs, their related Cas9-sensitive cells were significantly more than the Cas9-insensitive cells (*p* = 3.9e-11) (Fig. 1C). In contrast, the non-Cas9-miRNAs, such as miR-1-3p (Fig. 1D, F, H), miR-215-5p, and miR-340-5p (Fig. S1), did not show such a trend. We therefore suggest that Cas9 RNA may act as a microRNA sponge, specifically influencing Cas9-miRNA target genes.

Among all the Cas9-miRNAs, let-7i was the mainly expressed Cas9-miRNA in most cells (Fig. S2). As let-7 family members are well-known tumor suppressor miRNAs [13, 14], the possible “miRNA sponge” effect of Cas9 on this family is worthy of further study. We also found by analysis with miRanda software that Cas9 RNA can bind to many other let-7 family members (Fig. S3). Moreover, through qPCR and Western Blot experiments, we confirmed that some validated let-7 target genes were significantly upregulated after the introduction of Cas9 in more types of cells (U251, 786-O, T98G *v.s.* MCF7) (Fig. 1I and J, Fig. S4).

We then investigated why the microRNA sponge effect of Cas9 varies greatly among different cell lines. Again, we used let-7 as a representative microRNA. Based on a joint analysis of the transcriptome data of 331 cell lines and the basic miRNA and mRNA expression data and gene mutation data in the Cancer Cell Line Encyclopedia (CCLE) dataset, we identified the top 35 types of cells in which Cas9 induced the greatest increase in let-7i target genes as “let-7i-related Cas9-sensitive cells” and vice versa. We found that the basal expression of let-7 family members, especially let-7i, was lower in “let-7i-related Cas9-sensitive cells” rather than the control cells (Fig. 1K, Fig.S5). At the same time, the basal expression levels of let-7 target genes were also lower in “let-7i-related Cas9-sensitive cells” (Fig. 1L, Fig. S6). We also investigated the gene mutation frequency in the two groups of cells. Interestingly, we found almost all high-frequency mutations of let-7 target genes are distributed in the “let-7i-related Cas9-insensitive” cells (Fig. 1M, Fig. S7). These results suggested that, if some key let-7 target genes are mutated, the effect of Cas9 on up-regulating let-7 target genes might be weakened. All these data indicate that the sensitivity of cells to Cas9-mediated miRNA sponge effect may be determined by complicating factors, including the appropriate expression levels of Cas9-miRNAs and their target genes and the tumor mutation burden levels, especially for the miRNA target genes.

Next, we analyzed the miRNA sponge effect for more types of Cas9 RNA. We found that another popular Cas9 RNA used in our lab also had a very high possibility of binding the let-7 family, especially let-7b (Fig. S8A). Moreover, RNA pulldown experiments confirmed that let-7 could bind this Cas9 RNA directly (Fig. 1N). Moreover, Cas9 RNA was significantly decreased when let-7 mimics were introduced into HEK293-Cas9 cells stably expressing Cas9 (Fig. S8B). After Gene Expression Omnibus (GEO) analysis of the public dataset GSE84534 [2], we found that target genes of the let-7 family were positively enriched after introducing Cas9, which has the same mRNA sequence as the Cas9 in our laboratory (Fig. S8C-D). Several reported let-7 target genes were found to be upregulated in some tumor cells after this Cas9 introduction, such as CDK6, KRAS, FN1, and HMGA2 (Fig. 1O, S8E and S9A-B). However, the corresponding changes were not obvious after the introduction of excess let-7 (Fig. 1P), suggesting that Cas9 affects let-7 target genes through a “miRNA sponge” mechanism depending on the appropriate let-7 expression level, which is consistent with the results of previous bioinformatics analysis (Fig. 1K, Fig. S5). Furthermore, we found that dCas9-VP64, which was used for gene activation [15], could also increase the expression of some let-7 target genes in U251 cells (Fig. S9C).

Moreover, in Cas9-transgenic mice, KRAS and CDK6 expression levels were also found to be mildly increased in the testis tissues (Fig. 1Q), although they were not significantly changed in most of the tissues we tested (Fig. S10), which might also be associated with the basic expression levels of let-7 and its target genes.

These results suggest that Cas9 RNA could regulate let-7 target genes through the miRNA sponge mechanism in some types of cells, indicating that Cas9 itself may affect cells through mechanisms other than DNA cleavage.

## RNA sequence optimization of Cas9 to reduce its effect on cell proliferation and let-7 downstream genes

**Fig. 2:**
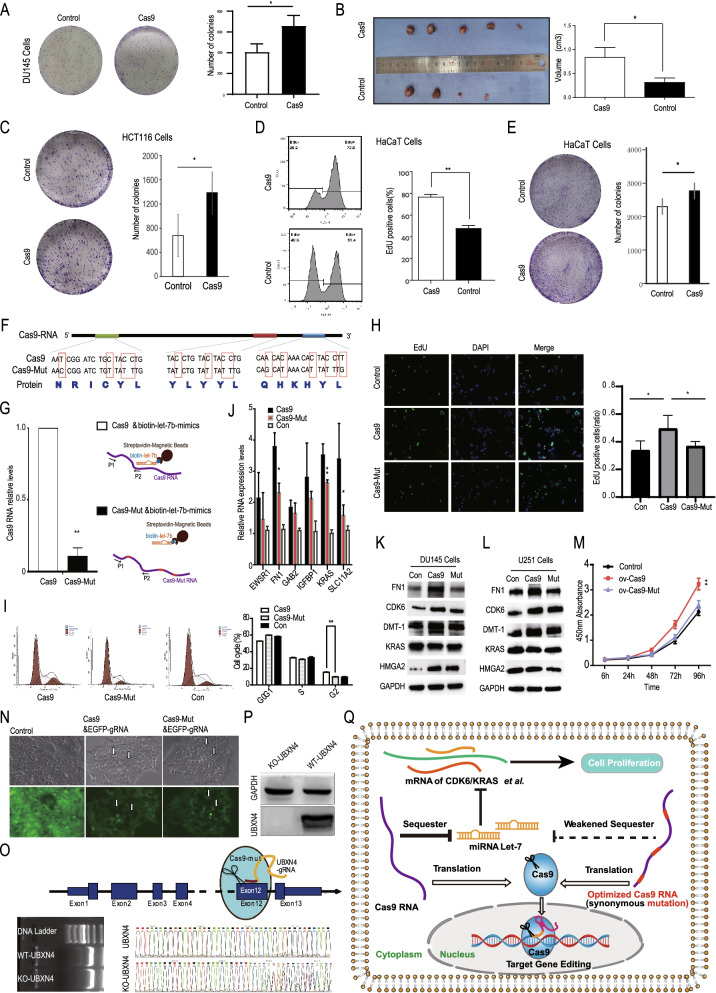
RNA sequence optimization of Cas9 to reduce its effect on cell proliferation and let-7 downstream genes. **A**, **C**, **E** Clonogenic assay showed an increased number of colonies formed after the introduction of Cas9 into DU145 (**A**), HCT116 (**C**), and HaCat (**E**) cells. The colonies were quantified by ImageJ software. Data are presented as the mean ± SD. *, *p* < 0.05, t test; *n* = 3. **B** Xenograft experiments showed increased growth of DU145 cells after the introduction of Cas9 in nude mice. The ruler scale is 0.5 mm per minor mark. *P < 0.05, t test. **D** An EdU assay showed an increased number of EdU-positive cell colonies formed after the introduction of Cas9 into HaCaT cells. The numbers of EdU-positive cell colonies were quantified by flow cytometry. Data are presented as the mean ± SD., *, *p* < 0.05, t test; *n* = 3. **F** Mononucleotide mutations were performed on several sites in Cas9 RNA that bind the let-7 family, resulting in an optimized Cas9-Mut RNA that was unable to bind Let-7. We transduced the Cas9 plasmid, Cas9-Mut plasmid, and control vector into cells for subsequent experiments. **G** RNA pull-down strategy and results in HEK293 cells. Biotin-labeled miR-let-7 mimic-transfected HEK293 cells were transfected with Cas9 or Cas9-mut expression plasmids. RNA was pulled down using streptavidin magnetic beads and then subjected to qPCR analysis. The data are presented as the mean ± SD. **, *p* < 0.01, t test, *n* = 3. **H** An EdU assay showed an increased number of EdU-positive cells after the introduction of Cas9 instead of Cas9-mut into DU145 cells. EdU-positive cells were quantified by ImageJ software. Data are presented as the mean ± SD. *, *p* < 0.05, ANOVA; *n* = 3. **I** The percentage of cells in cell cycle phases was determined by flow cytometry with propidium iodide (PI) staining. The results are shown as the mean ± SD. **, *p* < 0.01, ANOVA; *n* = 3. **J**-**K** Let-7 target genes were detected by qPCR (**J**) and Western blotting (**K**) in DU145 cells transduced with Cas9, Cas9-Mut or the control vector. The qPCR data are presented as the mean ± SD. *, *p* < 0.05, ANOVA; *n* = 3. **L** Let-7 target genes were detected by Western blotting in Cas9-, Cas9-Mut- or control vector-transduced U251 cells. **M** Cell proliferation was assessed after introduction of Cas9, Cas9-Mut or the control vector in HepG2 cells using a CCK-8 kit. **, *p* < 0.01, ANOVA, *n* = 3. **N** EGFP knockout cells were found after co-transfection of the Cas9-Mut plasmid and gRNA expression plasmid targeting EGFP in a cell line (HEK293) that stably expressed EGFP. **O** Strategy and results of knocking out an endogenous gene, UBXN4, using the Cas9-Mut plasmid. DU145 cells were transduced with gRNA targeting the UBXN4 gene and Cas9-Mut plasmids. UBXN4 gene knockout (KO) was confirmed by PCR. Sanger sequencing of the PCR product showed the emergence of an abnormal peak at the gRNA targeting site, which indicated successful UBXN4 knockout in some cells. **P** UBXN4 knockout by Cas9-Mut in DU145 cells was detected by Western blotting. GAPDH was used as a loading control. **Q** A schematic representation of the unexpected effects of Cas9 at RNA levels and our solution strategy. The widely used Cas9 RNA can bind and sequester let-7 through the miRNA sponge mechanism, thus upregulating let-7 target genes and slightly promoting cell proliferation. In our optimization strategy, synonymous mutation of Cas9, in which the binding site of let-7 was mutated, could weaken its role in promoting cell proliferation but still maintain its gene-editing function

The majority of let-7 downstream target genes are oncogenes [13, 14]. Since Cas9 can regulate the expression of some let-7 target genes, we speculate that exogenously introduced Cas9 may affect the biological characteristics of cells. We transduced the Cas9 viral vector, as well as the control virus, into the human prostate cancer cell line DU145 and found that Cas9 slightly promoted cell proliferation in a Cell Counting Kit-8 (CCK-8) experiment (Fig. S11A), cell cycle assay (Fig. S11B), and cell colony formation assay (Fig. 2A). In an in vivo experiment, we also found that the tumor growth rate and tumor volume were slightly higher in the group with stable Cas9 expression (Fig. 2B).

In addition, we introduced Cas9 into several other types of tumor cells, such as the human renal carcinoma cell line 786–O and the human colon carcinoma lines HT-29 and HCT116. Cas9 had no significant effect on HT-29 cells (data not shown) but slightly promoted the proliferation of HCT116 and 786–O cells (Fig. 2C, Fig. S11C). These results indicate that Cas9 does not have a significant effect on all cells but could slightly promote the proliferation of DU145 cells, 786–O cells and HCT116 cells.

Moreover, Cas9 could also increase some let-7 target genes in normal human cultured primary bone marrow mesenchymal stem cells (bMSCs) (Fig. S12 A-E) and the human normal epithelial cell line HaCaT (Fig. S12F-G) and could promote the proliferation of HaCaT cells (Fig. 2D-E).

In summary, we found that Cas9 RNA could slightly promote proliferation in some cells through a miRNA sponge mechanism. Although the effect is weak, especially in vivo*,* our observations suggested that it is necessary to improve the safety of CRISPR–Cas9 technology by modifying Cas9 itself in addition to reducing the off-target effect of the CRISPR–Cas9 system.

To further confirm the presence of this miRNA sponge mechanism and to optimize the Cas9 sequence at the RNA level, we constructed a synonymous mutant plasmid named Cas9-Mut. Cas9-Mut had mutations at the three let-7 binding sites; however, the mRNA transcript from Cas9-Mut could be translated into the same amino acid sequence as Cas9 (Fig. 2F). In fact, the RNA pulldown results in HEK293 cells showed that the binding of Cas9-Mut to let-7 was significantly reduced compared to that of Cas9 (Fig. 2G). Moreover, although Cas9-Mut somewhat promoted colony formation in DU145 cells compared with the control cells, its activity was weaker than that of Cas9 (Fig. S13). EdU assays showed that the proliferation ability of Cas9-Mut-transfected DU145 cells was weaker than that of Cas9-transfected cells (Fig. 2H). The percentage of G1 phase cells increased and that of G2 phase cells decreased in the Cas9-Mut group compared with the Cas9 group (Fig. 2I). At the molecular level, we found that the ability of Cas9-Mut to upregulate the let-7 target gene was also significantly weaker than that of Cas9, not only in DU145 cells (Fig. 2J-K) but also in U251 cells (Fig. 2L). Additionally, in hepatic carcinoma cell line HepG2, the cell proliferation ability of Cas9-Mut-transfected cells was weaker than that of Cas9-transfected cells (Fig. 2M). Although the current mutants cannot completely prevent the binding of let-7 and Cas9 RNA at other sites with higher binding free energy nor can they prevent the binding of other miRNAs that may affect proliferation, such as miR-145-5p, the partial effectiveness of the mutation suggests that Cas9 RNA can be further optimized in the future by the method of synonymous mutation.

Theoretically, because the amino acid sequence in Cas9-Mut is not changed, the ability to cleave DNA is not changed (Fig. 2F). To determine whether the optimized Cas9-Mut still has a gene-editing function, we first tested the ability of Cas9-Mut to knock out the EGFP reporter gene. After the introduction of Cas9-Mut and the designed EGFP-gRNA into HEK293 cell line cells stably expressing EGFP, some EGFP-negative cells appeared, indicating that Cas9-Mut still has a gene-editing function (Fig. 2N). We further tested the gene-editing ability of Cas9-Mut by transducing the Cas9-Mut plasmid and gRNA targeting the UBXN4 gene into DU145 cells (Fig. 2O). Genomic DNA was extracted, and the sequence around the predicted cutting site was amplified by PCR (Fig. 2O). Sanger sequencing of this PCR product showed double peaks around the predicted cutting site, which suggested successful DNA cutting (Fig. 2O). Furthermore, UBXN4 protein was not expressed in the selected single clones of DU145 cells by Western blotting (Fig. 2P). Therefore, the gene-editing function of Cas9-Mut was confirmed, providing support for its potential use in practical applications.

## Conclusion

Our bioinformatic analysis and experimental results suggest that Cas9 RNA could interact with endogenous miRNA through the miRNA sponge mechanism, and it is valuable to optimize CRISPR–Cas9 technology at the RNA level for safer application in the future. Specifically, the current widely used Cas9 RNA could bind and sequester let-7, thus upregulating some let-7 target genes and slightly promoting cell proliferation in some cell types. Through synonymous mutation, the RNA sequence optimization of Cas9, introducing modified let-7 binding sites, weakened the effect on the promotion of cell proliferation and the expression of some let-7 downstream genes (Fig. 2N).

## Supplementary Information

**Additional file 1: Table S1**. MiRNA List of “Cas9-miRNAs”. **Table S2**. MiRNA List of “Non-Cas9-miRNAs”. **Figure S1**. Cas9 RNA showed the trend of disturbing endogenous genes through “miRNA sponge” mechanism. **Figure S2**. Basic expression levels of 12 Cas9-miRNAs in different cell lines. **Figure S3**. The let-7 family predicted to bind the RNA of Cas9 that has been introduced into 331 cell lines. **Figure S4**. Experimental validation of the expression levels of some let-7 target genes in MCF7. **Figure S5**. Appropriate expression level of let-7 family, especially let-7i-5p, might be related to whether Cas9 regulates the target genes of let-7i-5p. **Figure S6**. Basic expression level of let-7 target genes might be related to whether Cas9 regulates these genes by miRNA sponge mechanism. **Figure S7**. Mutation of let-7 target genes were related to whether Cas9 regulates let-7 target genes by miRNA sponge mechanism. **Figure S8**. Cas9 regulates the targets of let-7 through a sponge mechanism. **Figure S9**. Cas9 and dCas9-VP64 promote the target genes of let-7 after transduction through adenovirus. **Figure S10**. Cas9 slightly upregulated the expression of let-7 downstream genes in limited tissue samples from Cas9-transgenic mice. **Figure S11**. Cas9 slightly promoted the proliferation of DU145 and 786-O cells. **Figure S12**. Cas9 regulates the target genes of let-7 through a sponge mechanism in bMSC and Hacat cells. **Figure S13**. RNA sequence optimization of Cas9 could reduce its effect on cell proliferation in DU145.

## Abbreviations

CRISPR: Clustered regularly interspaced short palindromic repeats
CDS: Coding sequences
gRNA: guide RNA
miRNA: microRNA
mRNA: Messenger RNA
GSEA: Gene set enrichment analysis
NES: Normalized enrichment score
AV: Adenovirus
qPCR: quantitative real-time PCR
CCK-8: Cell Counting Kit-8
HaCaT: Human normal epithelial cell line
bMSCs: Bone marrow mesenchymal stem cells
EGFP: Enhanced Green Fluorescent Protein
KO: Knockout
PCR: Polymerase Chain Reaction
DEN: Diethylnitrosamine
CCl4: Carbon tetrachloride
PFA: Paraformaldehyde
BSA: Bovine Serum Albumin
DAPI: 4,6-diamino-2-phenyl indole
cDNA: Complementary DNA
CCLE: Cancer Cell Line Encyclopedia

## References

[1] Zhang H (2021). Application of the CRISPR/Cas9-based gene editing technique in basic research, diagnosis, and therapy of cancer. Mol Cancer.

[2] Chew WL (2016). A multifunctional AAV-CRISPR-Cas9 and its host response. Nat Methods.

[3] Charlesworth CT (2019). Identification of preexisting adaptive immunity to Cas9 proteins in humans. Nat Med..

[4] Kleinstiver BP (2016). High-fidelity CRISPR-Cas9 nucleases with no detectable genome-wide off-target effects. Nature.

[5] Tay Y (2008). MicroRNAs to Nanog, Oct4 and Sox2 coding regions modulate embryonic stem cell differentiation. Nature.

[6] Zhang K (2018). A novel class of microRNA-recognition elements that function only within open reading frames. Nat Struct Mol Biol.

[7] Hansen TB (2013). Natural RNA circles function as efficient microRNA sponges. Nature.

[8] Chen D-L (2021). The circular RNA circDLG1 promotes gastric cancer progression and anti-PD-1 resistance through the regulation of CXCL12 by sponging miR-141-3p. Mol Cancer.

[9] Wang Y (2013). Endogenous miRNA sponge lincRNA-RoR regulates Oct4, Nanog, and Sox2 in human embryonic stem cell self-renewal. Dev Cell.

[10] Xiao G (2019). The long noncoding RNA TTTY15, which is located on the Y chromosome, promotes prostate Cancer progression by sponging let-7. Eur Urol..

[11] Xu C (2016). Long non-coding RNA GAS5 controls human embryonic stem cell self-renewal by maintaining NODAL signalling. Nat Commun.

[12] Enache OM (2020). Cas9 activates the p53 pathway and selects for p53-inactivating mutations. Nat Genet.

[13] Powers JT (2016). Multiple mechanisms disrupt the let-7 microRNA family in neuroblastoma. Nature.

[14] Busch B (2016). The oncogenic triangle of HMGA2, LIN28B and IGF2BP1 antagonizes tumor-suppressive actions of the let-7 family. Nucleic Acids Res.

[15] Dai W (2019). Cancer therapy with a CRISPR-assisted telomerase-activating gene expression system. Oncogene..

