# Supplementary material for: Optimization of Cas9 RNA sequence to reduce its unexpected effects as a microRNA sponge
Source: Mol Cancer. 2022 Jun 24;21:136. doi: 10.1186/s12943-022-01604-x (PMC9229757; doi:10.1186/s12943-022-01604-x)
Supplement: Supplementary file 1 — Additional file 1: Table S1. MiRNA List of “Cas9-miRNAs”. Table S2. MiRNA List of “Non-Cas9-miRNAs”. Figure S1. Cas9 RNA showed the trend of disturbing endogenous genes through “miRNA sponge” mechanism. Figure S2. Basic expression levels of 12 Cas9-miRNAs in different cell lines. Figure S3. The let-7 family predicted to bind the RNA of Cas9 that has been introduced into 331 cell lines. Figure S4. Experimental validation of the expression levels of some let-7 target genes in MCF7. Figure S5. Appropriate expression level of let-7 family, especially let-7i-5p, might be related to whether Cas9 regulates the target genes of let-7i-5p. Figure S6. Basic expression level of let-7 target genes might be related to whether Cas9 regulates these genes by miRNA sponge mechanism. Figure S7. Mutation of let-7 target genes were related to whether Cas9 regulates let-7 target genes by miRNA sponge mechanism. Figure S8. Cas9 regulates the targets of let-7 through a sponge mechanism. Figure S9. Cas9 and dCas9-VP64 promote the target genes of let-7 after transduction through adenovirus. Figure S10. Cas9 slightly upregulated the expression of let-7 downstream genes in limited tissue samples from Cas9-transgenic mice. Figure S11. Cas9 slightly promoted the proliferation of DU145 and 786-O cells. Figure S12. Cas9 regulates the target genes of let-7 through a sponge mechanism in bMSC and Hacat cells. Figure S13. RNA sequence optimization of Cas9 could reduce its effect on cell proliferation in DU145. [file 12943_2022_1604_MOESM1_ESM.pdf]

Supplementary Information

Optimization of Cas9 RNA sequence to reduce its unexpected effects as a microRNA sponge

Authors

Junfeng Jiang*[,1,2], Tao Zeng*[,1,2,3], Li Zhang*[4], Xingfei Fan*[,1,2], Qishu Jin[5], Haitao Ni[1], Yusheng Ye[1], Lipeng Cheng[1], Li Li[1], Liujun Wang[1], Sha Xu[1], Yu Yang[1], Juan Gu[1], Bing Guo[6,7,8], Lei Wang[6,7,8], Xin Li[6,7,8], Yingyi Qin[9], Jiaxi Li[10], Jinjiang Wang[10], Xi Chen[11], Minjuan Wu[1], Qi-long Ying[§,11], Xingjun Qin[§, 6,7,8], Yefei Wang[§,12,13], Yue Wang[§, 1,2]

Affiliations

1: Histology and Embryology Department, Naval Medical University, Shanghai, 200433, China

2: Shanghai Key Laboratory of Cell Engineering, Naval Medical University, Shanghai, 200433, China.

3: The 901th Hospital of PLA Jiont Logistic Support Force, HeFei, 230031, China

4: Department of Pathology, Faculty of Medical Imaging, Naval Medical University, Shanghai, 200433, China

5: Department of Histology and Embryology, Harbin Medical University, Harbin 150086, China

6: Department of Oral & Maxillofacial - Head & Neck Oncology, Shanghai Ninth People’s Hospital, College of Stomatology，Shanghai Jiao Tong University School of Medicine, Shanghai, 200011, China

7: National Clinical Research Center for Oral Diseases, Shanghai, 200011, China

8: Shanghai Key Laboratory of Stomatology, Shanghai, 200011, China

9: Department of health statistics, Naval Medical University, Shanghai, 200433, China

10: Department of Oncology, Tongren Hospital, Shanghai Jiao Tong University School of Medicine, Shanghai, 200336, China

11: Department of Cell and Neurobiology, Eli and Edythe Broad Center for Regenerative Medicine and Stem Cell Research at USC, Keck School of Medicine, University of Southern California, Los Angeles, California 90033, USA

12: Department of Ophthalmology, Ninth People’s Hospital, Shanghai Jiao Tong University School of Medicine, Shanghai, 200011, China

13: Shanghai Key Laboratory of Orbital Diseases and Ocular Oncology, Shanghai, 200011, China

Contact

Prof. Yue Wang

Histology and Embryology Department, Naval Medical University, 800 Xiangyin Road, Shanghai, 200433, China.

Tel and Fax: +86-21-81870964. Email: wangyuesmmu@163.com

Prof. Yefei Wang

Department of Ophthalmology, Ninth People's Hospital, Shanghai Jiao Tong University School of Medicine, Shanghai, 200011, China, E-mail: paper34@163.com

Prof. Xingjun Qin

Shanghai Ninth People's Hospital, Shanghai Jiao Tong University School of Medicine, Shanghai Key Laboratory of Stomatology, 639 Zhi Zao Ju Road, Shanghai, 200011, China, Email: qinxj1989@sina.com

Prof. Qi-long Ying

Eli and Edythe Broad Center for Regenerative Medicine and Stem Cell Research at USC, Keck School of Medicine, University of Southern California, Los Angeles, California 90033, USA, Email: qying@med.usc.edu

Running Title

Optimization of Cas9 RNA sequence to improve its safety

Highlights

Cas9 RNA functions as a miRNA sponge.

Let-7 is the dominant regulated miRNA by Cas9 RNA.

RNA sequence optimization of Cas9 by synonymous mutation improves its safety.

Keywords

CRISPR–Cas9, miRNA sponge, Let-7, RNA sequence optimization

---

* These authors contribute equally to this work.

§ Corresponding authors

1. Supplementary Tables

Table. S1 MiRNA List of "Cas9-miRNAs"

MiRNA List of Cas9-miRNAs (Totally 51 miRNAs)				
hsa-let-7i-5p	hsa-miR-145-5p	hsa-miR-494-5p	hsa-miR-6813-5p	hsa-miR-3619-5p
hsa-miR-1180-3p	hsa-miR-1908-5p	hsa-miR-5009-5p	hsa-miR-6824-5p	hsa-miR-3691-5p
hsa-miR-12126	hsa-miR-1915-5p	hsa-miR-5195-3p	hsa-miR-6875-3p	hsa-miR-3692-5p
hsa-miR-1238-3p	hsa-miR-3065-3p	hsa-miR-6075	hsa-miR-7156-5p	hsa-miR-3944-3p
hsa-miR-1249-3p	hsa-miR-3120-5p	hsa-miR-6514-5p	hsa-miR-7706	hsa-miR-431-3p
hsa-miR-1250-5p	hsa-miR-4472	hsa-miR-663a	hsa-miR-7843-5p	hsa-miR-4435
hsa-miR-1268a	hsa-miR-4640-5p	hsa-miR-6721-5p	hsa-miR-3183	hsa-miR-4440
hsa-miR-1268b	hsa-miR-4660	hsa-miR-6746-3p	hsa-miR-3185	
hsa-miR-1289	hsa-miR-4741	hsa-miR-6774-5p	hsa-miR-320a-5p	
hsa-miR-1293	hsa-miR-4743-3p	hsa-miR-6786-5p	hsa-miR-329-5p	

The Cas9 RNA sequence is based on pLX311-Cas9[1]， Addgene #118018. The 51 "Cas9-miRNAs" are those that can be predicted to bind Cas9 RNA under strict standards in miRanda software: the value of "score" parameter is higher than 160, and the value of " energy" parameter is lower than -25 kCal/Mol.

Table. S2 MiRNA List of "Non-Cas9-miRNAs"

MiRNA List of Non-Cas9-miRNAs (Totally 69 miRNAs)				
hsa-miR-1-3p	hsa-miR-335-5p	hsa-miR-4503	hsa-miR-548ah-3p	hsa-miR-5688
hsa-miR-12135	hsa-miR-340-5p	hsa-miR-4509	hsa-miR-548ah-5p	hsa-miR-5692b
hsa-miR-1252-5p	hsa-miR-3613-3p	hsa-miR-4643	hsa-miR-548aj-3p	hsa-miR-5700
hsa-miR-1468-3p	hsa-miR-3618	hsa-miR-4645-5p	hsa-miR-548am-3p	hsa-miR-577
hsa-miR-181b-3p	hsa-miR-3668	hsa-miR-4662a-3p	hsa-miR-548ap-3p	hsa-miR-580-5p
hsa-miR-190a-3p	hsa-miR-374a-5p	hsa-miR-4677-3p	hsa-miR-548aq-3p	hsa-miR-590-3p
hsa-miR-190b-3p	hsa-miR-374b-5p	hsa-miR-4680-3p	hsa-miR-548ar-5p	hsa-miR-606
hsa-miR-2054	hsa-miR-374c-5p	hsa-miR-4760-3p	hsa-miR-548av-3p	hsa-miR-607
hsa-miR-215-5p	hsa-miR-3910	hsa-miR-4770	hsa-miR-548c-3p	hsa-miR-633
hsa-miR-3117-5p	hsa-miR-3942-3p	hsa-miR-4782-3p	hsa-miR-548f-3p	hsa-miR-656-3p
hsa-miR-3123	hsa-miR-4262	hsa-miR-4801	hsa-miR-548j-3p	hsa-miR-8084
hsa-miR-3140-5p	hsa-miR-4272	hsa-miR-5007-3p	hsa-miR-548x-3p	hsa-miR-9-3p
hsa-miR-3145-5p	hsa-miR-4311	hsa-miR-548ad-3p	hsa-miR-559	hsa-miR-98-3p
hsa-miR-3201	hsa-miR-4434	hsa-miR-548ae-3p	hsa-miR-568	

The 69 "Non-Cas9-miRNAs" are those that couldn't be predicted to bind Cas9 RNA under loose standards in miRanda software: the value of "score" parameter is lower than 60, and the value of " energy" parameter is higher than -5 kCal/Mol.

2. Supplementary Figures

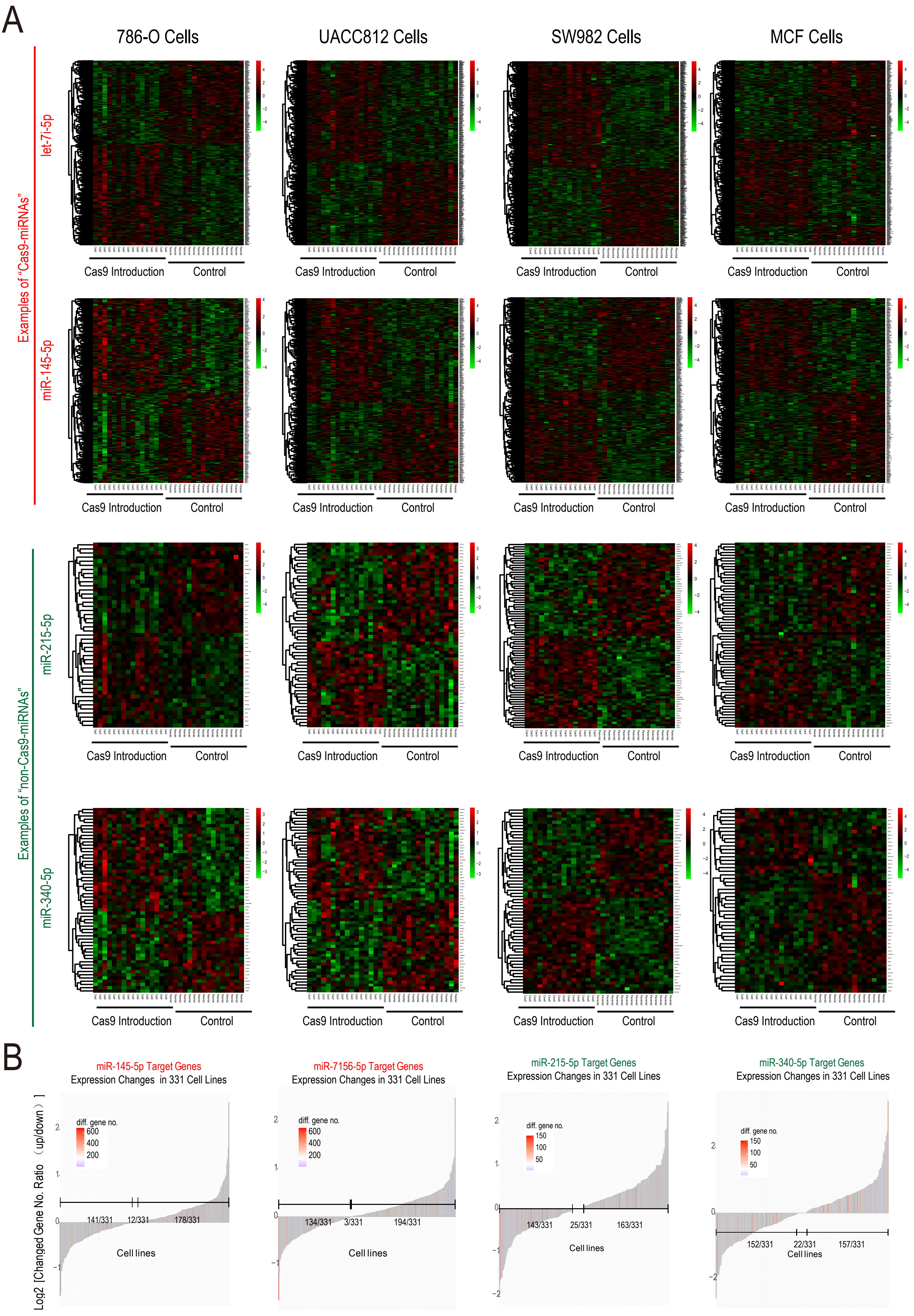
A
786-O Cells
UACC812 Cells
SW982 Cells
MCF Cells
Examples of "Cas9-miRNAs"
let-7i-5p
miR-145-5p
Examples of "non-Cas9-miRNAs"
miR-215-5p
miR-340-5p
Cas9 Introduction
Control
B
miR-145-5p Target Genes
Expression Changes in 331 Cell Lines
141/331
12/331
178/331
miR-7156-5p Target Genes
Expression Changes in 331 Cell Lines
134/331
3/331
194/331
miR-215-5p Target Genes
Expression Changes in 331 Cell Lines
143/331
25/331
163/331
miR-340-5p Target Genes
Expression Changes in 331 Cell Lines
152/331
22/331
157/331
diff. gene no.
Cell lines
Log2 [Changed Gene No. Ratio （up/down）]

Fig. S1 Cas9 RNA showed the trend of disturbing endogenous genes through "miRNA sponge" mechanism

(A) Heatmaps of the expression levels of target genes of let-7i, miR-145-5p, miR-215-5p and miR-340-5p before or after Cas9 introduction in 4 other cell type examples. Let-7i and miR-145-5p were two examples of Cas9-miRNAs. MiR-215-5p and miR-340-5p were two examples of non-Cas9-miRNAs.

(B) Expression changes of target genes of 2 Cas9-miRNAs examples and 2 non-Cas9-miRNAs examples in 331 cell lines. MiR-145-5p and miR-7156-5p are examples of 51 "Cas9-miRNAs" which have been predicted to bind Cas9 RNA. MiR-215-5p and miR-340-5p are examples of 69 "non-Cas9-miRNAs" which could not bind to Cas9 RNA by prediction based on sequences. Each bar represents the situation in one cell type, the value of the bar is the log2 value of the ratio of up-regulated gene number to down-regulated gene number.

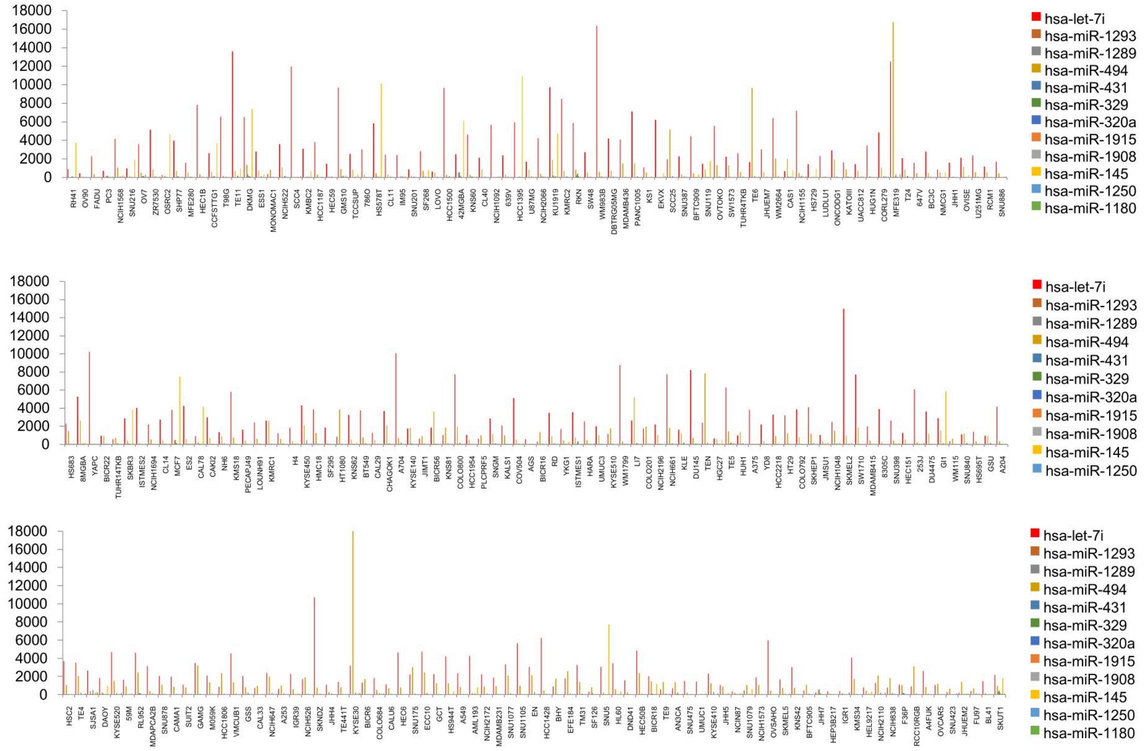

Fig. S2 Basic expression levels of 12 Cas9-miRNAs in different cell lines

After joint analysis of the 331 cell lines data and their basic miRNA expression data in Cancer Cell Line Encyclopedia (CCLE, https://sites.broadinstitute.org/ccle）, we found that 12 of the 51 "Cas9-miRNAs" can be found in CCLE date set, and 239 of the 331 cell lines can be found in CCLE date set. The bar graph showed the basic expression of these 12 miRNAs in these 239 cell lines. This result showed that let-7i is the main expressed miRNA in most cells, miR-145 also showed highly expressed in most cells, the other Cas9-miRNAs show very low expression in most cells when compared to let-7i.

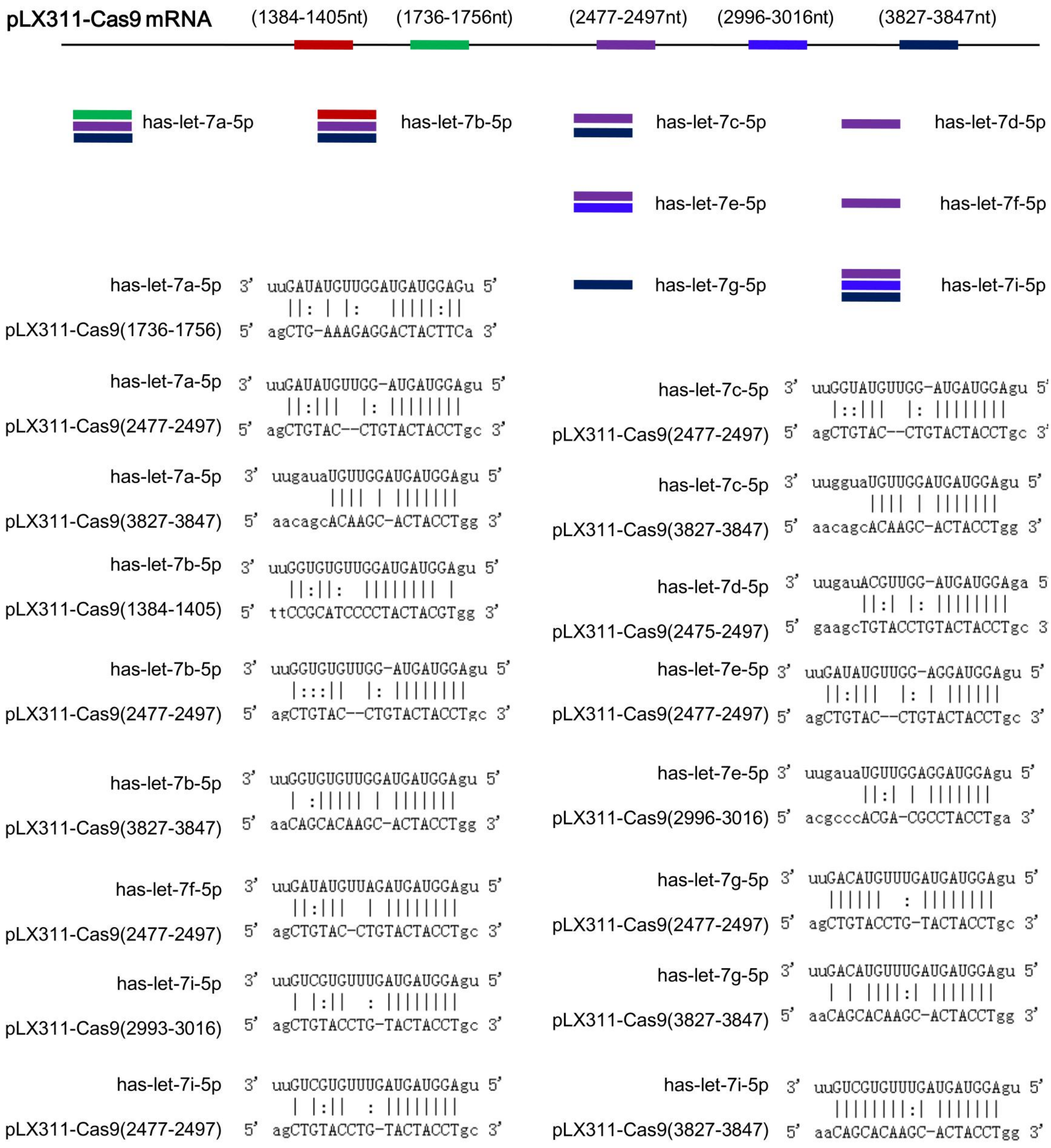

Fig. S3 The let-7 family predicted to bind the RNA of Cas9 that has been introduced into 331 cell lines

Illustration and miRanda results showing the miRNA binding sites of the let-7 family in the full-length of the Cas9 RNA sequence predicted using the miRanda tool (this Cas9 has been introduced into 331 cell lines).

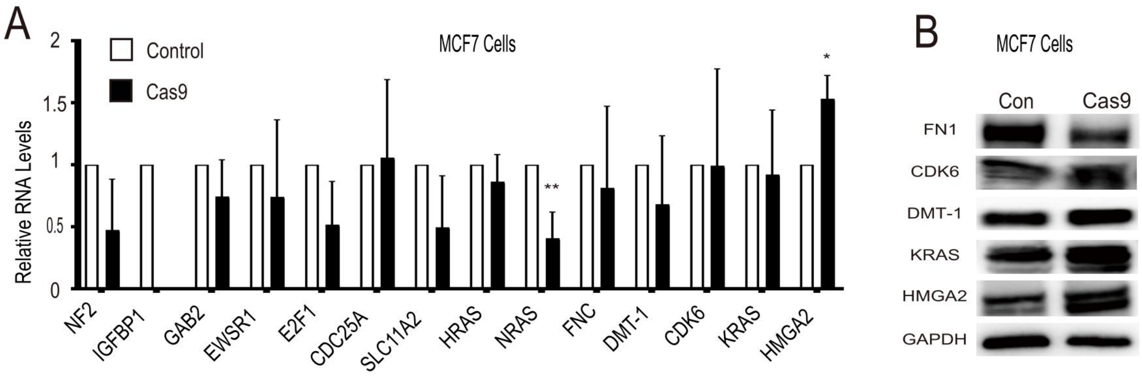

Fig. S4 Experimental validation of the expression levels of some let-7 target genes in MCF7.

qPCR and Western blot analysis showed changes in the levels of representative downstream genes of let-7 after infection of lentivirus expression this Cas9 that has been induced in the 331 cell lines. The data are presented as the means ± SD. *, $p<0.05$, t test; n=3.

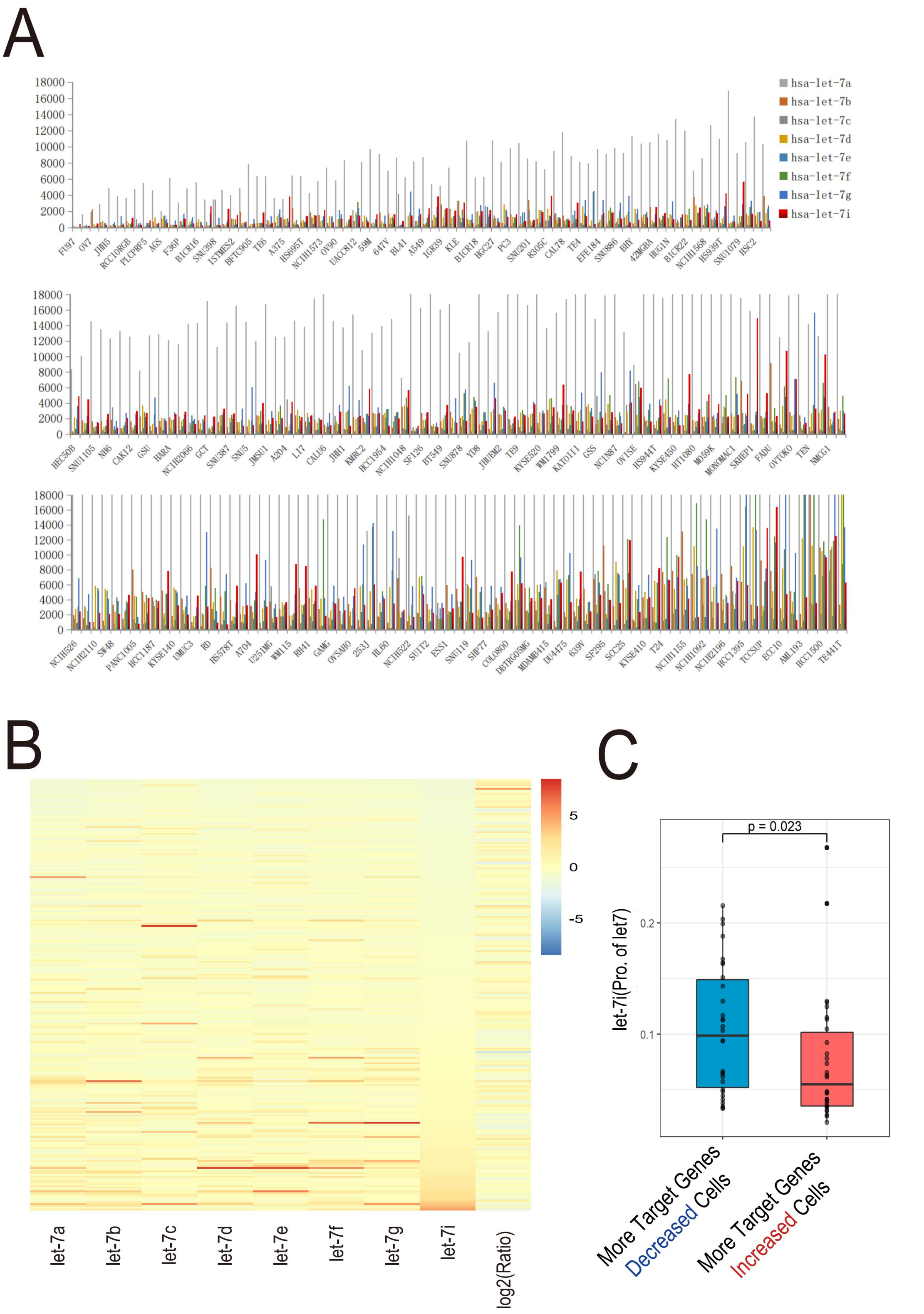

Fig. S5 Appropriate expression level of let-7 family, especially let-7i-5p, might be related to whether Cas9 regulates the target genes of let-7i-5p

(A) Basic expression levels of let-7 family in 239 of the 331 cell lines based on Cancer Cell Line Encyclopedia (CCLE).

(B) Heatmap of the expression levels of let-7 family and the log2(Ratio) in all the 239 cell lines which has miRNA expression data in CCLE. log2(Ratio): log2 value of the ratio of upregulated gene number to downregulated gene number, which is the same number in Fig.1E. From top to bottom, the cells were arranged from low to high according to the let-7i expression levels. The heatmap showed that higher log2(Ratio) often appears in lower let-7, especially let-7i expressing cell types.

(C) Visual display the let-7i expression proportion of all let-7 family in two cell groups. More Target Genes Increased cells: the 186 types of cells in which more let-7i target genes were increased than decreased after Cas9 introduction. More Target Genes Decreased cells: the 139 cell types of cells in which more let-7i target genes were decreased than increased after Cas9 introduction.

These data showed that Cas9 RNA can better play the let-7i sponge role on up-regulating let-7i target genes in cells with relatively low let-7i expression. These results were reasonable, because if there are too many let-7 in the cells, the sequester effect of Cas9 RNA on let-7i will be insignificant. Considering the basic expression levels of let-7 family are very high in most cells as shown in Figure S2, we believe that the appropriate expression level of let-7 family, especially let-7i-5p, might be related to whether Cas9 regulates these target genes of let-7i-5p.

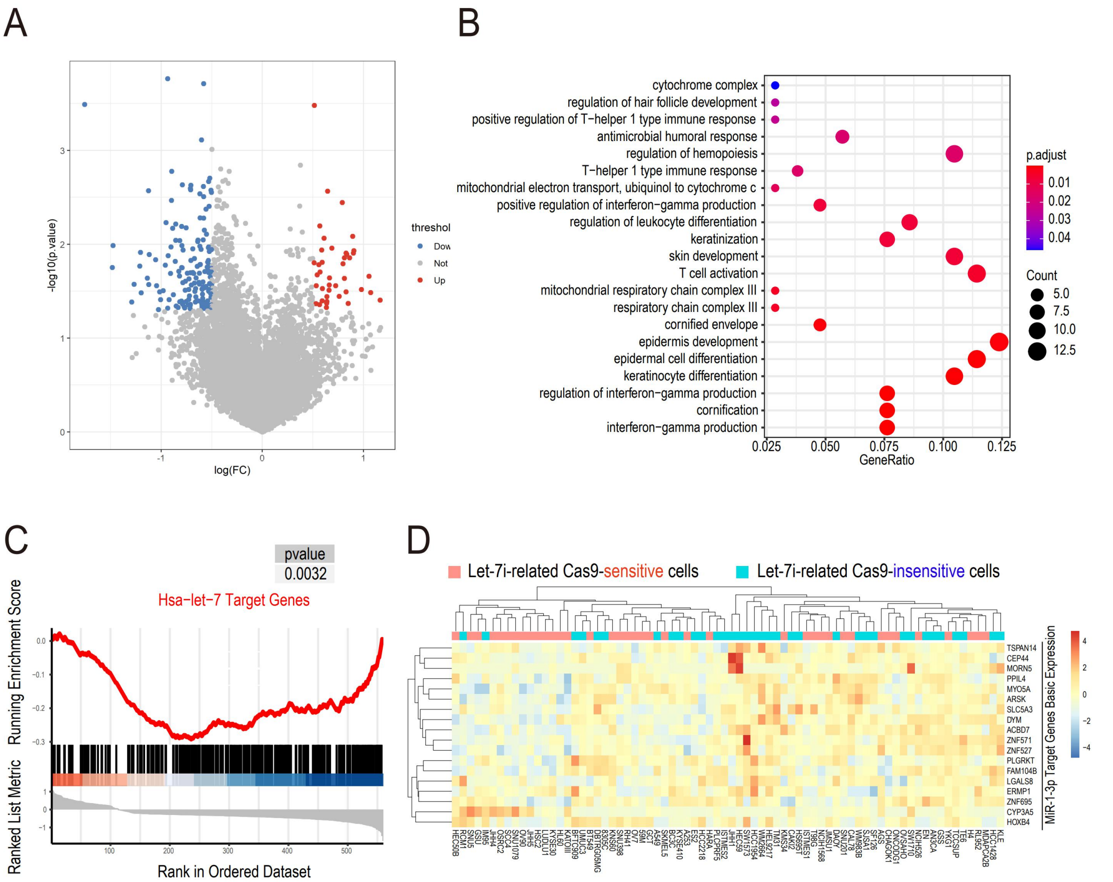

Fig. S6 Basic expression level of let-7 target genes might be related to whether Cas9 regulates these genes by miRNA sponge mechanism

(A) Volcano chart showed the different basic expression genes between " Let-7i-related Cas9-sensitive cells" and " Let-7i-related Cas9-insensitive cells". Let-7i-related Cas9-sensitive cells: the top 35 types of cells in which more let-7i target genes were increased than decreased after Cas9 introduction. Let-7i-related Cas9-insensitive cells: the top 35 cell types in which more let-7i target genes were decreased than increased after Cas9 introduction. The basic gene expression levels in each cell line were mined from CCLE. These data showed there were some genes that have different basal expression levels between the " Let-7i-related Cas9-sensitive cells" and " Let-7i-related Cas9-insensitive cells"

(B) GO analysis result of the different basic expression genes between " Let-7i-related Cas9-sensitive cells" and " Let-7i-related Cas9-insensitive cells". This data showed genes that have different basal expression levels between the two groups are not very significantly enriched in a specific pathway.

(C) GSEA analysis of all the validated let-7 target genes based on the different gene basic expression data between " Let-7i-related Cas9-sensitive cells" and " Let-7i-related Cas9-insensitive cells". And let-7 target genes were found to be negatively enriched in " Let-7i-related Cas9-sensitive cells".

(D) Heatmap showed the differently expressed target genes of miR-1-3p between "Let-7i-related Cas9-sensitive cells" and "Let-7i-related Cas9-insensitive". It showed there was no significant difference of the basic expression of these mir-1-3p target genes between the two groups of cells.

These data suggested that the basic expression levels of let-7 target genes are also related to whether they are regulated by Cas9, and if the expression level of target genes is too high, it is unlikely to further significantly up regulate such target genes through miRNA sponge mechanism.

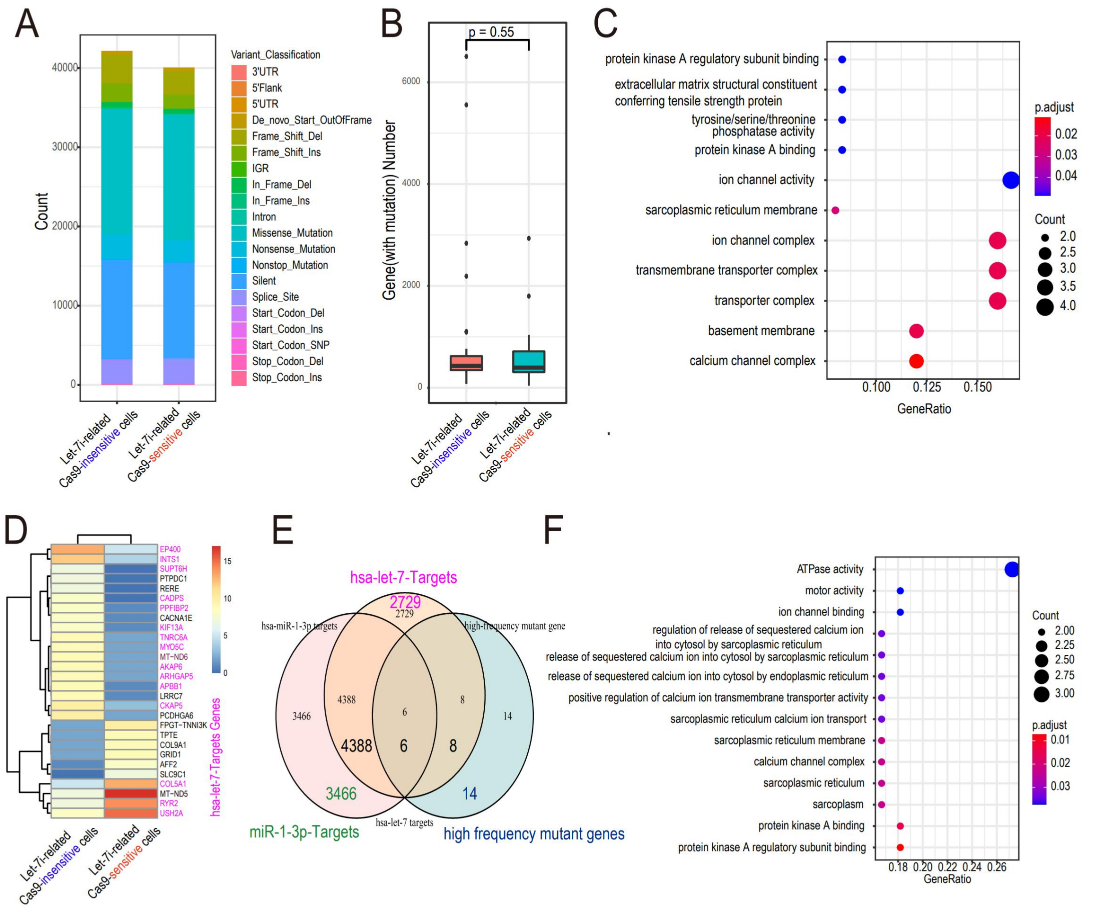

Fig. S7 Mutation of let-7 target genes were related to whether Cas9 regulates let-7 target genes by miRNA sponge mechanism

(A) There was no big difference in mutation events distribution between " Let-7i-related Cas9-sensitive cells" and " Let-7i-related Cas9-insensitive cells".

Let-7i-related Cas9-sensitive cells: the top 35 types of cells in which more let-7i target genes were increased than decreased after Cas9 introduction.

Let-7i-related Cas9-insensitive cells: the top 35 cell types in which more let-7i target genes were decreased than increased after Cas9 introduction. The mutation data in each cell line were mined from CCLE.

(B) There was no big difference in mutated Gene number between " Let-7i-related Cas9-sensitive cells" and " Let-7i-related Cas9-insensitive cells".

(C) GO analysis of the 28 high-frequency mutant genes in all the 70 cell lines. Those whose mutation frequency is higher than 10% are considered to be high-frequency mutations. This data showed high-frequency mutant genes in the two groups are not significantly enriched in a specific pathway.

(D) Heatmap showed the number distribution of the 28 high-frequency mutant genes in the " Let-7i-related Cas9-insensitive cells" and " Let-7i-related Cas9-sensitive cells". The names of the reported let-7 target genes were marked purple.

(E) Venn diagram showed that among the 28 high-frequency mutant genes in the " Let-7i-related Cas9-insensitive cells" and " Let-7i-related Cas9-sensitive cells", half of the genes (14) are let-7 target, which is higher than the number (6) of the target genes of mirna-1-3p (a non-Cas9-miRNA).

(F) GO analysis of the 14 let-7 target genes that are also high-frequency mutant genes in all the 70 cell lines.

This result is very interesting, because almost all let-7 target genes with high-frequency mutations are distributed in the " Let-7i-related Cas9-insensitive cells". These results suggested that, if some key let-7 target genes are mutated, the effect of Cas9 on up regulating let-7 target genes will be weakened.

On the other hand, in the " Let-7i-related Cas9-sensitive cells", there was almost no mutation distribution of these high-frequency mutant let-7 target genes, suggesting

that if Cas9 is to play the role of up regulating let-7 target genes, some key let-7 target genes could not be mutated.

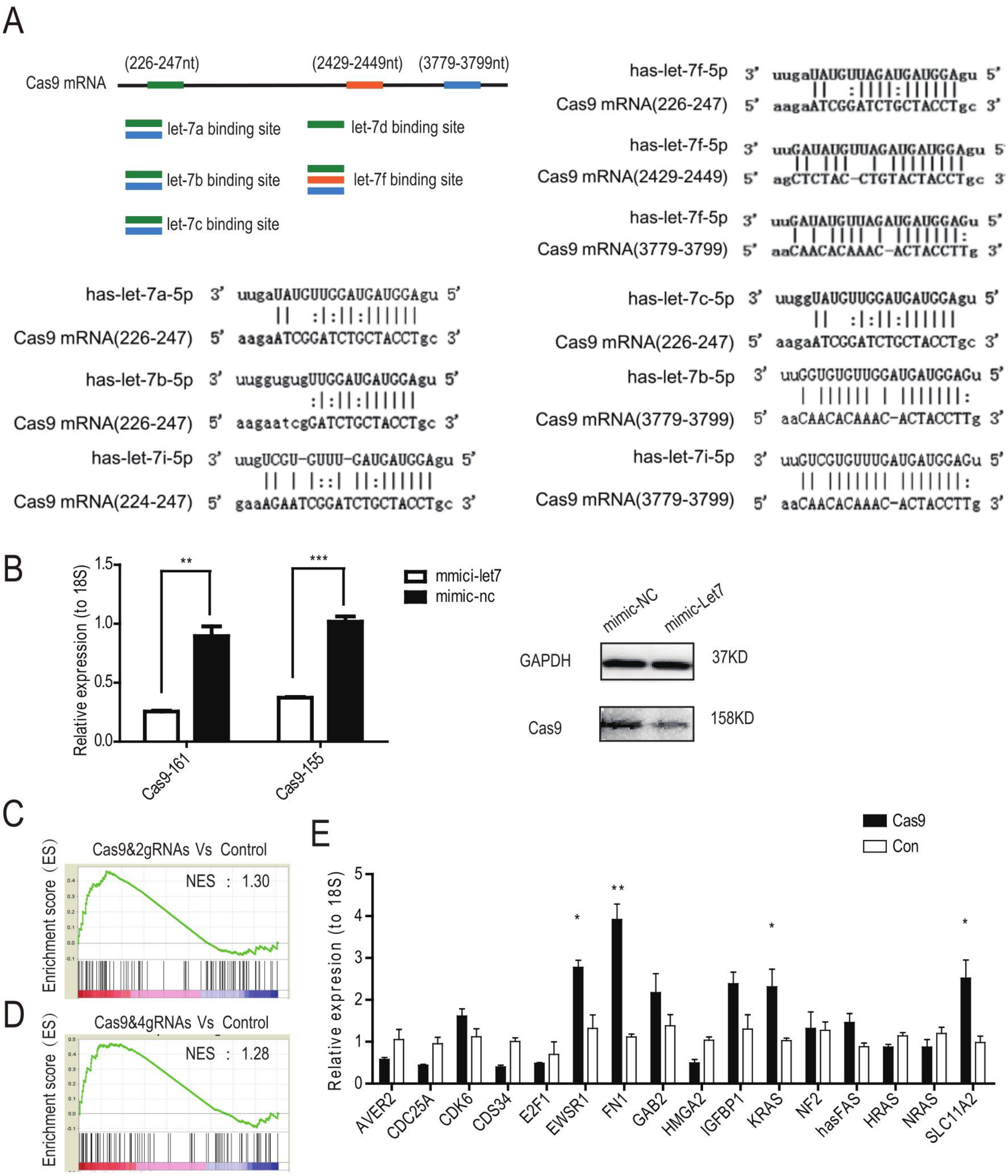

Fig. S8 Cas9 regulates the targets of let-7 through a sponge mechanism

(A) Illustration and miRanda results showing the miRNA binding sites of the let-7 family in the full-length Cas9 RNA sequence predicted using the miRanda tool (based on plasmid #41815 from Addgene, this Cas9 RNA has been used in our laboratory).

(B) Cas9 levels were repressed after transfection of let-7 mimics RNAs into the HEK293-Cas9 cell line, which stably expresses Cas9. Protein expression levels were assayed with Western blot analysis, and the mRNA levels were assayed by qPCR analysis using two pairs of primers. The data are presented as the mean ± SD. **, $p<0.01$, t test, n=3.

(C-D) GSEA of all the validated let-7 target genes listed in the miRTarBase database from the GSE84534 dataset. The gene expression data were from the muscle tissue after infection of AAV9-Cas9 (and gRNAs targeting Mstn and Fst) or Control AAV9 in (C) and from the muscle tissue after infection of AAV9-Cas9 (and gRNAs targeting Mstn, Fst, Pd-l1, Cd47) or Control AAV9 in (D). Let-7 target genes were found to be positively enriched after introducing Cas9. NES: normalized enrichment score.

(E) Let-7 target genes were selected from published datasets. The mRNA levels of these genes were detected by qPCR in Cas9- or control vector-transduced DU145 cells. The data are presented as the mean ± SD. **, $p<0.01$; t test, n=3.

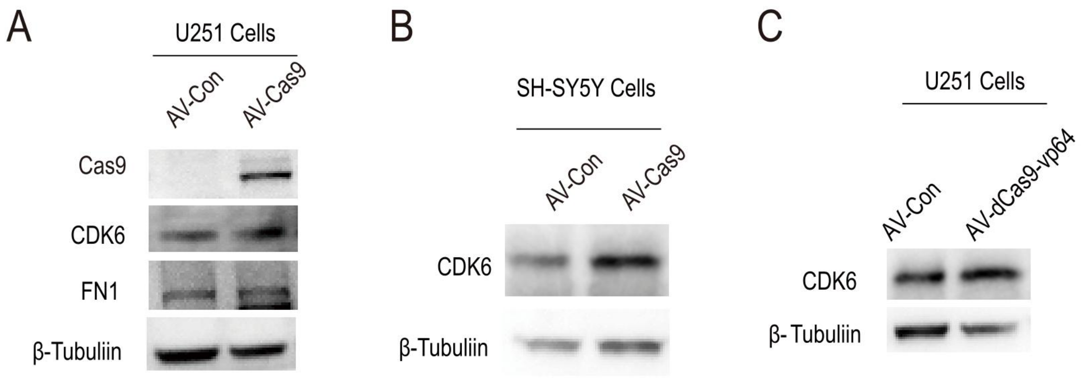

Fig. S9 Cas9 and dCas9-VP64 promote the target genes of let-7 after transduction through adenovirus

Western blot analysis of the protein expression levels of representative let-7 target genes after Cas9 introduction through Cas9-expression- adenovirus. The protein levels of some let-7 targets were increased after introducing Cas9 (A-B) and dCas9-VP64(C) adenovirus transduction. AV: adenovirus. U251: a human glioma cell line. SH-SY5Y: a human neuroblastoma cell line. dCas9-VP64: mutated Cas9 for gene activation, the let-7 binding sites are not mutated.

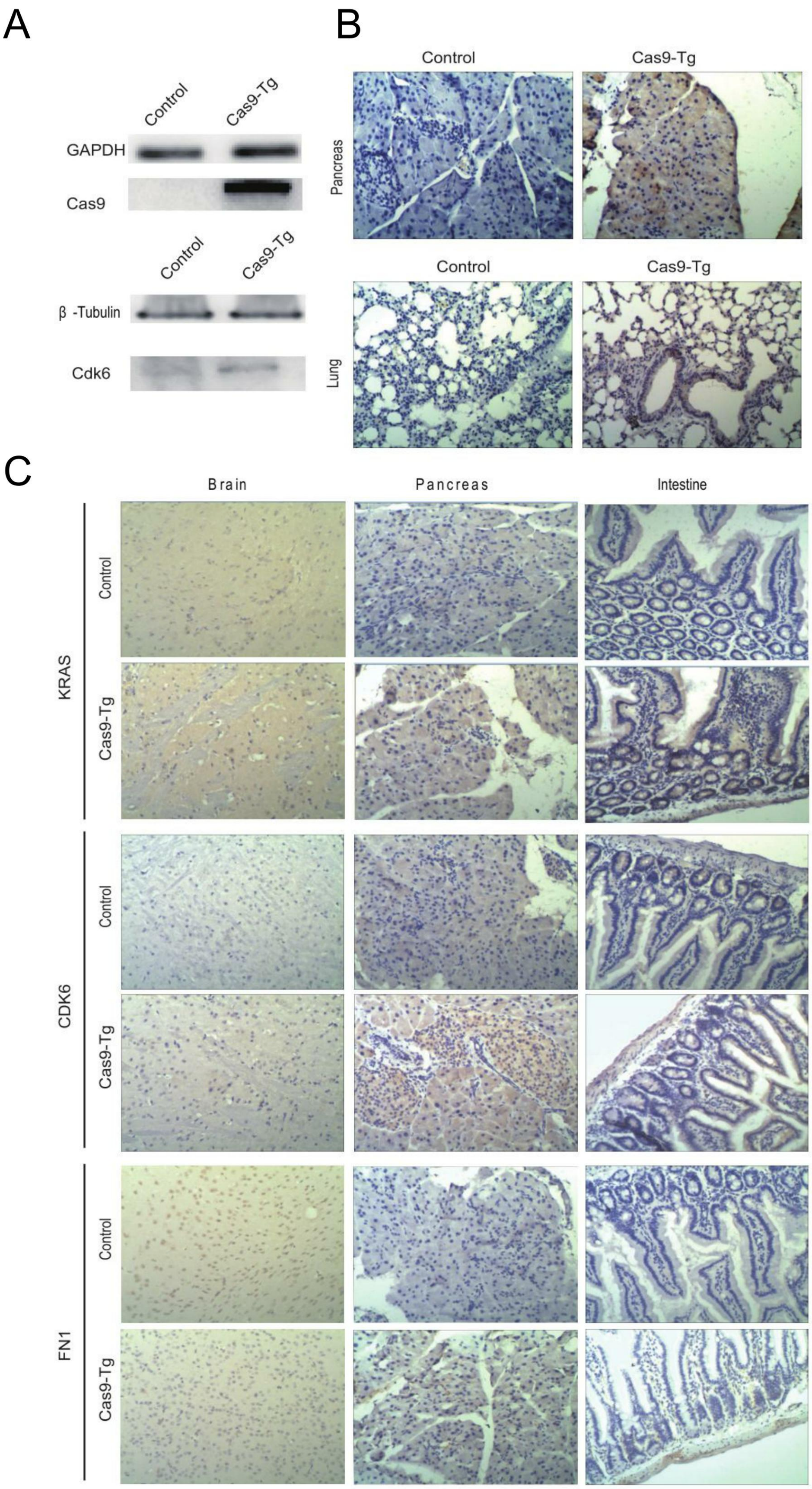

Fig. S10 Cas9 slightly upregulated the expression of let-7 downstream genes in

limited tissue samples from Cas9-transgenic mice

(A) Protein levels of Cas9 and CDK6 in the tail tissues of Cas9-transgenic and control mice analyzed by Western blotting.

(B) Immunohistochemistry analysis of Cas9 expression in pancreas and lung tissues of Cas9-transgenic mice and control mice. Cas9-Tg: Cas9-transgenic mouse.

(C) Immunohistochemistry analysis of KRAS, CDK6 and FN1 protein levels in tissues from the brain, pancreas and intestine of Cas9-transgenic mice and control mice. We also analyzed these genes in kidney, liver, lung, stomach, spine and bladder tissues of Cas9-transgenic and control mice and they were not significantly changed （data not shown）. Cas9-Tg: Cas9-transgenic mouse.

In these *in vivo* experiments, we did not find strong evidence that Cas9 has a significant effect on mice. This may be due to the complexity of the organism and compensatory regulation, but it is worthy of more systematic and in-depth research in the future. For example, it is valuable to investigate whether there is a higher incidence of spontaneous cancer or any other disorder in their lifespan. Or a certain sample size of Cas9 transgenic mice may be exposed to tumorigenic factors, such as irradiation, diethylnitrosamine (DEN) or carbon tetrachloride (CCl_4), and test whether Cas9-Tg mice have a higher tumor incidence than normal mice. Such experiments are worth pursuing in follow-up studies.

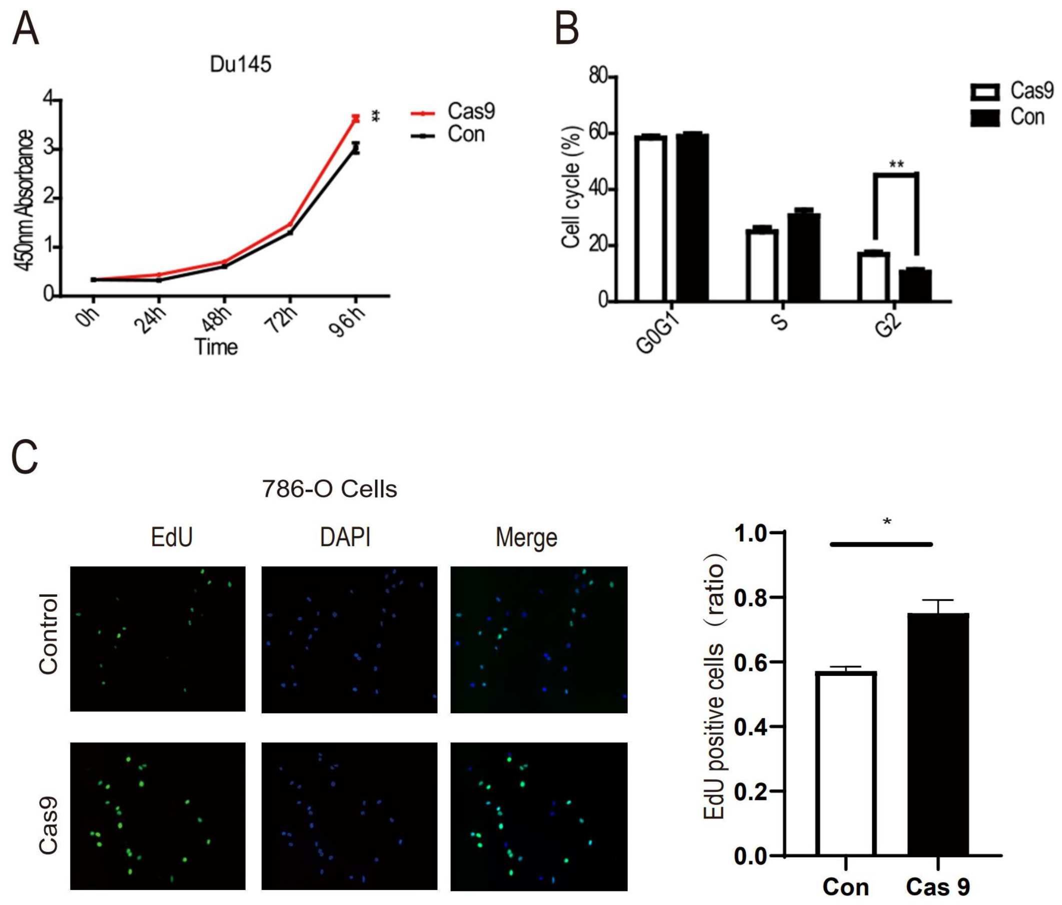

Fig. S11 Cas9 slightly promoted the proliferation of DU145 and 786-O cells

(A) Cell proliferation was assessed after the introduction of Cas9 into DU145 cells using CCK-8. *, $p<0.05$, and **, $p<0.01$, t test; n=3.

(B) The cell cycle was determined by flow cytometry (BD) with PI staining. Values are presented as the mean ± SD. **$P<0.01$, t test.

(C) EdU assay showed an increased number of EdU-positive cells after the introduction of Cas9 into 786-O cells. EdU-positive cells were quantified by ImageJ software. Data are presented as means ± SD. *$p < 0.05$, t test; n = 3.

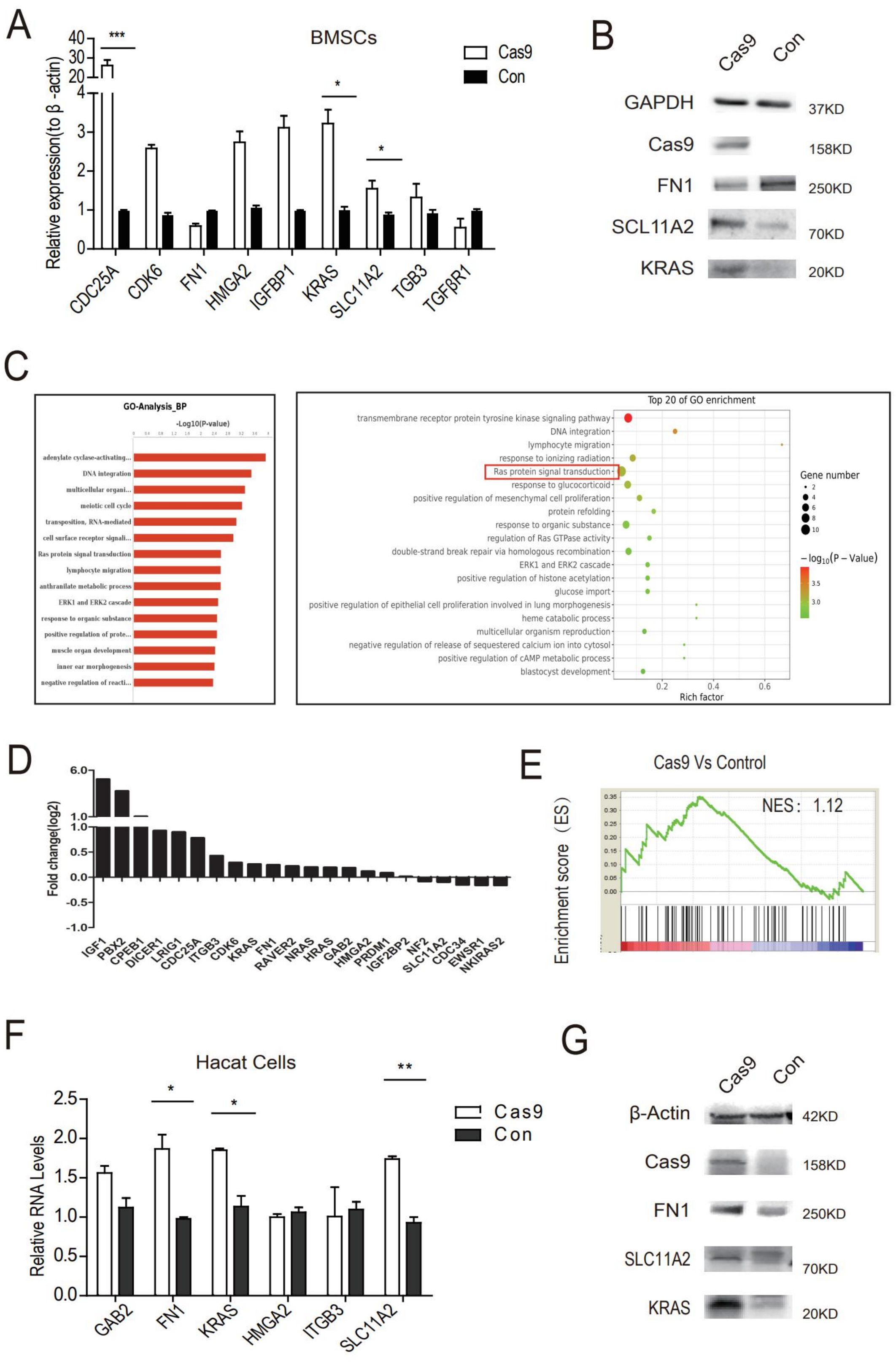

Fig. S12 Cas9 regulates the target genes of let-7 through a sponge mechanism in bMSC and Hacat cells

(A-B) qPCR and Western blot analysis show changes in the levels of representative downstream genes of let-7 after transfection of Cas9 and control plasmids into bMSCs. 18S was used as the normalization control in qPCR analysis. The data are presented as the means ± SD. **, $p<0.01$, t test, n=3.

(C) GO enrichment analysis of expression genes tested by high throughput RNA sequencing after introduction of Cas9 or the control vector in bMSCs. The data show that genes involved in the Ras signaling pathway and mesenchymal cell proliferation were enriched.

(D) RNA expression levels of representative let-7 target genes in bMSCs after introduction of Cas9 or the control vector based on high throughput RNA sequencing.

(E) GSEA of all the let-7 target genes tested by high-throughput RNA sequencing after introduction of Cas9 or the control vector in bMSCs showed positive enrichment (NES:1.12) after introduction of Cas9. NES: normalized enrichment score.

(F-G) qPCR and Western blot analysis show changes in the levels of representative downstream genes of let-7 after transfection of Cas9 and control plasmids into Hacat cells. 18S was used as the normalization control in qPCR analysis. The data are presented as the means ± SD. **, $p<0.01$, t test, n=3.

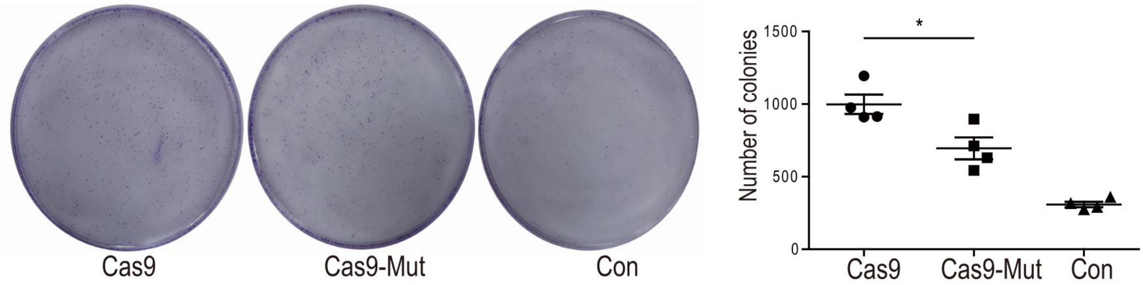

Fig. S13 RNA sequence optimization of Cas9 could reduce its effect on cell proliferation in DU145

Clonogenic assay of DU145 cells after introduction of Cas9, Cas9-Mut or the control vector. The colonies of the cells were quantified by ImageJ software. Data are presented as the mean ± SD. *$p < 0.05$, t test; $n = 3$.

3. Supplementary Materials and methods

3.1 Cell Culture.

DU145 and 786-O cells were cultured in RPMI1640(Sigma) containing 10% fetal bovine serum (FBS, Gibco). HEK293 cells, T98G, HCT116 and U251 cells were cultured in DMEM plus 10% FBS (Gibco, Grand Island, NY, USA), Penicillin (100 U/mL) / Streptomycin (100 μg/mL). SH-SY5Y cells were cultured in Dulbecco's Modified Eagle's Medium: Ham's Nutrient Mixture F-12, 1:1 (D-MEM/F-12) (Gibco, Grand Island, NY, USA) supplemented with 10% FBS, Penicillin (100 U/mL) / Streptomycin (100 μg/mL) in a humidified incubator at 37°C and 5% CO2. Bone marrow-derived mesenchymal stem cells (BMSCs) were cultured in Mesenchymal Stem Cell Basal Medium (MSCBM, DAKEWE) with 5% Cell Culture Supplement, EliteGro™-Adv (EliteCell #EPA-050). All of the cells placed in a 37°C incubator with 5% CO2.

3.2 Knock-out of genes using CRISPR/Cas9-Mut

To investigate whether Cas9-Mut has a gene editing function, we constructed a HEK293 cell line stably expressing EGFP. We next constructed a gRNA expression vector targeting EGFP using AGCGTGTCCGGCGAGGGCG *AGG* as the binding sequence, which was located downstream of the U6 promoter. To target the endogenous UBXN4 gene, CAAGGTCCAAATGTCTCCGC *TGG* was designed for gRNA binding. The gRNA expression vector was co-transduced with Cas9 or Cas9-Mut into targeting cells. Single clones were picked and cultured in 96-well plates. DNA samples were extracted using a QIAamp DNA Micro Kit (QIAGEN) according to the manufacturer's instructions.

3.3 Cell Counting Kit-8 (CCK-8) Assay

Transfected cells were counted and seeded into 96-well plates at a density of 10,000 cells/well. Detection of cell proliferation was performed after 24h. Cell proliferation was detected by a CCK-8 assay (Beyotime, China) according to the instructions of manufacturer. A 10 μL volume of CCK-8 assay solution and an 100uL volume of medium were added to wells prior to incubation at 37°C for 1h in a cell incubator. After the incubation, the 450nm absorbance of each well was determined by an Infinite 200 reader (TECAN). Each sample was performed in triplicate.

3.4 Cell Cycle Analysis

For cell cycle analysis, Cell Cycle and Apoptosis Analysis Kit (Beyotime, C1052, China) was used. Approximately $5x10^5$ cells were collected and fixed in cold 75% alcohol at least 4 hours, and then incubated with 500ul of PI solution at 37℃ under dark condition for 30 min. About $1x10^4$ cells were counted each time.

3.5 EdU Assay

Cells were trypsinized and added to the 96-well plates at a density of 2000 cells per well. After 24h, EdU kit (RIBOBIO, China) was used to measure cell proloferation according to the manufacturer's instructions.

3.6 Clonogenic Assays.

Cells were trypsinized and resuspended by 1mL of medium and then equal number of cells in each group were seeded in a well of 6-wells plate. To form the colonies, the cells were cultured in incubator for 8-10 days. Secondly, the colonies were washed by PBS three times, and fixed with 4% PFA about 20 min. After fixation, they were

stained with 0.1% crystal violet (Sigma) about 30 min. Finally, the colonies were washed by PBS and counted.

3.7 Cell Transfection and Infection

Cultured cells were transfected with plasmid by Lipofectamine 3000 (Invitrogen), according to the manufacturer's instructions. After 24 hours for transfection, the mixture medium was replaced with fresh medium. For the introduction of Cas9 or Cas9-VP64 into cells by virus, we got the Cas9-expression lenti-virus or adenovirus from Obio Technology (Shanghai) or Genechem Technology (Shanghai). The usage of the virus was in accordance with the company's instructions carefully.

3.8 RNA Isolation and Quantitative Real-time PCR Analysis

Total RNA of cells was extracted by Trizol reagent (Invitrogen), and the complementary DNA (cDNA)were synthesized by the PrimeScript™RT reagent Kitwith gDNA Eraser (Takara, RR047A). according to the manufacturer's instructions. For qPCR, FastStart Universal SYBR Green Master(ROX) was utilized, and 18S RNA or GAPDH mRNA was used as the endogenous control.

3.9 Western Blot

The total proteins of the cells were extracted by Whole Cell Lysis Assay (KeyGEN BioTECH, China) according to instructions of manufacturer. SDS–PAGE was done in 8–10%Tris-Glycine Gels (Invitrogen) and transferred to polyvinylidene fluoride (PVDF) membranes (Millipore). The membranes were blocked in 5% BSA, incubated with primary and secondary antibodies, and washed according to standard procedures. Primary antibodies were rabbit polyclonal anti-spCas9 (1:1000, Abways), rabbit polyclonal anti-KRAS (1:1000, Proteintech), rabbit monoclonal anti-HMGA2

（1:1000, Proteintech）, rabbit polyclonal anti-CDK6(1:1000, Proteintech), rabbit polyclonal anti-SLC11A2(also named DMT-1,1:1000, Proteintech), rabbit polyclonal anti-FN1(1:1000, Proteintech), rabbit polyclonal anti-GAPDH (1:2000,Biosharp).

3.10 Bioinformatics analysis

The binding possibilities between Cas9 RNA and all the known human miRNAs were analyzed using miRanda software. The database with gene expression levels of 331 Cas9-introduced cell lines and their parental control counterparts can be downloaded from the following website: https://clue.io/data/XPR_BASE#CAS9_BASELINE. The differentially expressed genes in each cell were determined by t test.

The Cas9-miRNAs were those that could be predicted to bind Cas9 RNA （pLX311-Cas9 [1], Addgene #118018, which has been used in the 331 cells dataset） under strict standards: the value of the score parameter was higher than 160, and the value of the energy parameter was lower than -25 kCal/Mol. The non-Cas9-miRNAs were those that could not be predicted to bind Cas9 RNA under loose parameters standards: threshold = 60, energy = -5kcal / mol, gap open penalty = - 8, gap extend penalty = -2; from all the 2656 miRNAs downloaded from miRBase database, 2587 miRNAs contained gene binding sites in these loose parameters, and "No hit“” was found on the other 69 miRNAs. These 69 miRNAs were the non-Cas9-miRNAs.

The target genes of each miRNA were determined by the union of the prediction results of TargetScan and miRanda using the default parameter: gap open penalty:

-9.000000， Gap Extend Penalty: -4.000000， Score Threshold: 140.000000， Energy Threshold: -14.000000 kcal/mol， Scaling parameter: 4.000000.

The basic mRNA and miRNA expression data and the gene mutation data of 239 cell lines were mined in Cancer Cell Line Encyclopedia (CCLE, https://sites.broadinstitute.org/ccle ） .The GEO dataset GSE84534 was used to compare the mRNA expression levels between groups transduced with Cas9 (with gRNAs) or the control and was analyzed for all the let-7 target genes using GSEA. The target genes of the let-7 miRNA family were found in miRTarBase with validation by at least two experimental methods and were used to construct the let-7 downstream geneset for GSEA of the GSE84534 dataset.

3.11 Animal experiments

The effect of Cas9 on the growth of tumors was determined using a xenograft model in male nude mice following reported protocols[12].Each nude mouse received 1×10^7 cells in 100 μL serum-free medium blended with an equal volume of BD Matrigel Matrix (BD Biosciences). Cas9-transgenic mice were obtained from Shanghai Research Center for Southern Model Organisms. The serum general biochemical indicators in Cas9-transgenic mice and normal control mice were tested with an automatic biochemical analyzer. All animal experiments were performed following National Institutes of Health guidelines for animal treatment with approval from the animal subjects committee at Naval Medical University.

3.12 RNA-pulldown analysis

The RNA-pulldown analysis was performed using an immunoprecipitation kit (RNA-binding protein immunoprecipitation–assay kit for microRNA; MBL, Nagoya, Japan).In brief, HEK293 cells were transfected with biotin-labled-miR-let7-mimic or biotin-labled-miR-NC-mimic， and infected Cas9-expression or Cas9-mut-expression lentivirus for 48 hours. Different groups of cell lysate with RNA were pulled down using Streptavidin Magnetic Beads (MCE, USA). The streptavidin-bound RNA was extracted following the manufacturer's instructions and then subjected to qPCR analysis.

3.13 Statistical analysis

The data are presented as the means ± SD. Statistical comparisons between the experimental groups were analyzed using ANOVA or two-tailed Student's t test for the data obtained from the qPCR analyses, clonogenic analyses， EdU analyses and gene expression assessment in each of the 331 cell types, and serum biomarkers assessment with an automatic biochemical analyzer and the luciferase activity assays. $P<0.05$ or $P<0.01$ was considered to indicate statistical significance.

3.16 Sequences

Primers Sequences

Gene		Sequence
GAPDH	F	GGCTGTTGTCATACTTCTCATGG
	R	GGAGCGAGATCCCTCCAAAAT
β -actin	F	GTGATCTCCTTCTGCATCCTGT
	R	CCACGAAACTACCTTCAACTCC
18S	R	TGGCTAGGACCTGGCTGTAT
	F	GTGGGCCGAAGATATGCTCA
Cas9-rt-161	F	TACGCCGGATACATTGACGG
	R	TGGGGGATGCTTCCATTGTC
Cas9-rt-155	F	GGACAGTCTTCACGAGCACA
	R	TTTGGTTCTCTCGGGCCATC
FN1	R	CGACCACATAGGAAGTCCCAG
	F	GAGAATAAGCTGTACCATCGCAA
KRAS	R	TTTCACACAGCCAGGAGTCTT
	F	ACAGAGAGTGGAGGATGCTTT
CDK6	R	TGAGGTTAGAGCCATCTGGAAA
	F	TCTTCATTCACACCGAGTAGTGC
TGFBR1-2	R	CCAATGGAACATCGTCGAGCA
	F	CACAGAGTGGGAACAAAAAGGT
ITGB3	R	GTCACCTGGTCAGTTAGCGT
	F	AGTAACCTGCGGATTGGCTTC
CDC34	R	GAGTATGGGTAGTCGATGGGG
	F	GACGAGGGCGATCTATACAACT
IGF2BP1	R	CCAGGGATCAGGTGAGACTG

	F	GGCCATCGAGAATTGTTGCAG
NF2	R	CAAGAAGTGAAAGGTGACTGGTT
	F	TTGCGAGATGAAGTGGAAAGG
NRAS	R	GGGCTTGTTTTGTATCAACTGTC
	F	CAGGGAGCAGATTAAGCGAGT
HRAS	R	GGCACGTCTCCCCATCAATG
	F	ATGACGGAATATAAGCTGGTGGT
HMGA2	R	CCTCTTGGCCGTTTTTCTCCA
	F	ACCCAGGGGAAGACCCAAA
CDC25A	R	TCGGTTGTCAAGGTTTGTAGTTC
	F	TTCCTCTTTTTACACCCCAGTCA
GAB2	R	CTGGGCGTCTTGAAGGTGTA
	F	ACAGTACCTACGACCTCCCC
EWSR1	R	GTGCATATCCTTGAGTGGGCT
	F	ATGGCGTCCACGGATTACAG
IGF2BP1	R	CCAGGGATCAGGTGAGACTG
	F	GGCCATCGAGAATTGTTGCAG
E2F1	R	CATCGATCGGGCCTTGTTTG
	F	CGTGTCAGGACCTTCGTAGC
SLC11A2	R	AGGATGACTCGTGGGACCTT
	F	CTAGACTGGGAGTGGTTACTGG
UBXN4-cut-12	F	AACCTTGGGCGTGATGTGAT
	R	AGAGGTGAAGCTGCTTTCCA

MiRNA-mimics Sequences

MiRNA-mimics		Sequence
hsa-let-7b-5p mimics	F	UGAGGUAGUAGGUUGUGUGGUU

	R	AACCACACAACCUACUACCUCA
mimics nc	F	UCACAACCUCCUAGAAAGAGUAGA
	R	UCUACUCUUUCUAGGAGGUUGUGA

Cas9 Sequence in pLX311-Cas9

This Cas9 has been introduced into 331 cell lines[1] to generate the big data we downloaded at following web site: https://clue.io/data/XPR_BASE#CAS9_BASELINE.

Additionally, the Cas9-transgenic mice we used also express this Cas9 sequence.

>pLX311-Cas9

ATGGCCCCAAAGAAGAAGCGGAAGGTCGGTATCCACGGAGTCCCAGCAG

CCGACAAGAAGTACAGCATCGGCCTGGACATCGGCACCAACTCTGTGGGC

TGGGCCGTGATCACCGACGAGTACAAGGTGCCCAGCAAGAAATTCAAGGT

GCTGGGCAACACCGACCGGCACAGCATCAAGAAGAACCTGATCGGAGCC

CTGCTGTTCGACAGCGGCGAAACAGCCGAGGCCACCCGGCTGAAGAGAA

CCGCCAGAAGAAGATACACCAGACGGAAGAACCGGATCTGCTATCTGCA

AGAGATCTTCAGCAACGAGATGGCCAAGGTGGACGACAGCTTCTTCCACA

GACTGGAAGAGTCCTTCCTGGTGGAAGAGGATAAGAAGCACGAGCGGCA

CCCCATCTTCGGCAACATCGTGGACGAGGTGGCCTACCACGAGAAGTACC

CCACCATCTACCACCTGAGAAAGAAACTGGTGGACAGCACCGACAAGGC

CGACCTGCGGCTGATCTATCTGGCCCTGGCCCACATGATCAAGTTCCGGG

GCCACTTCCTGATCGAGGGCGACCTGAACCCCGACAACAGCGACGTGGAC

AAGCTGTTCATCCAGCTGGTGCAGACCTACAACCAGCTGTTCGAGGAAAA

CCCCATCAACGCCAGCGGCGTGGACGCCAAGGCCATCCTGTCTGCCAGAC

TGAGCAAGAGCAGACGGCTGGAAAATCTGATCGCCCAGCTGCCCGGCGA

GAAGAAGAATGGCCTGTTCGGAAACCTGATTGCCCTGAGCCTGGGCCTGA

CCCCCAACTTCAAGAGCAACTTCGACCTGGCCGAGGATGCCAAACTGCAG

CTGAGCAAGGACACCTACGACGACGACCTGGACAACCTGCTGGCCCAGAT

CGGCGACCAGTACGCCGACCTGTTTCTGGCCGCCAAGAACCTGTCCGACG

CCATCCTGCTGAGCGACATCCTGAGAGTGAACACCGAGATCACCAAGGCC

CCCCTGAGCGCCTCTATGATCAAGAGATACGACGAGCACCACCAGGACCT

GACCCTGCTGAAAGCTCTCGTGCGGCAGCAGCTGCCTGAGAAGTACAAAG

AGATTTTCTTCGACCAGAGCAAGAACGGCTACGCCGGCTACATTGACGGC

GGAGCCAGCCAGGAAGAGTTCTACAAGTTCATCAAGCCCATCCTGGAAAA

GATGGACGGCACCGAGGAACTGCTCGTGAAGCTGAACAGAGAGGACCTG

CTGCGGAAGCAGCGGACCTTCGACAACGGCAGCATCCCCCACCAGATCCA

CCTGGGAGAGCTGCACGCCATTCTGCGGCGGCAGGAAGATTTTTACCCAT

TCCTGAAGGACAACCGGGAAAAGATCGAGAAGATCCTGACCTTCCGCATC

CCCTACTACGTGGGCCCTCTGGCCAGGGGAAACAGCAGATTCGCCTGGAT

GACCAGAAAGAGCGAGGAAACCATCACCCCCTGGAACTTCGAGGAAGTG

GTGGACAAGGGCGCTTCCGCCCAGAGCTTCATCGAGCGGATGACCAACTT

CGATAAGAACCTGCCCAACGAGAAGGTGCTGCCCAAGCACAGCCTGCTGT

ACGAGTACTTCACCGTGTATAACGAGCTGACCAAAGTGAAATACGTGACC

GAGGGAATGAGAAAGCCCGCCTTCCTGAGCGGCGAGCAGAAAAAGGCCA

TCGTGGACCTGCTGTTCAAGACCAACCGGAAAGTGACCGTGAAGCAGCTG

AAAGAGGACTACTTCAAGAAAATCGAGTGCTTCGACTCCGTGGAAATCTC

CGGCGTGGAAGATCGGTTCAACGCCTCCCTGGGCACATACCACGATCTGC

TGAAAATTATCAAGGACAAGGACTTCCTGGACAATGAGGAAAACGAGGA

CATTCTGGAAGATATCGTGCTGACCCTGACACTGTTTGAGGACAGAGAGA

TGATCGAGGAACGGCTGAAAACCTATGCCCACCTGTTCGACGACAAAGTG

ATGAAGCAGCTGAAGCGGCGGAGATACACCGGCTGGGGCAGGCTGAGCC

GGAAGCTGATCAACGGCATCCGGGACAAGCAGTCCGGCAAGACAATCCT

GGATTTCCTGAAGTCCGACGGCTTCGCCAACAGAAACTTCATGCAGCTGA

TCCACGACGACAGCCTGACCTTTAAAGAGGACATCCAGAAAGCCCAGGTG

TCCGGCCAGGGCGATAGCCTGCACGAGCACATTGCCAATCTGGCCGGCAG

CCCCGCCATTAAGAAGGGCATCCTGCAGACAGTGAAGGTGGTGGACGAG

CTCGTGAAAGTGATGGGCCGGCACAAGCCCGAGAACATCGTGATCGAAAT

GGCCAGAGAGAACCAGACCACCCAGAAGGGACAGAAGAACAGCCGCGA

GAGAATGAAGCGGATCGAAGAGGGCATCAAAGAGCTGGGCAGCCAGATC

CTGAAAGAACACCCCGTGGAAAACACCCAGCTGCAGAACGAGAAGCTGT

ACCTGTACTACCTGCAGAATGGGCGGGATATGTACGTGGACCAGGAACTG

GACATCAACCGGCTGTCCGACTACGATGTGGACCATATCGTGCCTCAGAG

CTTTCTGAAGGACGACTCCATCGACAACAAGGTGCTGACCAGAAGCGACA

AGAACCGGGGCAAGAGCGACAACGTGCCCTCCGAAGAGGTCGTGAAGAA

GATGAAGAACTACTGGCGGCAGCTGCTGAACGCCAAGCTGATTACCCAGA

GAAAGTTCGACAATCTGACCAAGGCCGAGAGAGGCGGCCTGAGCGAACT

GGATAAGGCCGGCTTCATCAAGAGACAGCTGGTGGAAACCCGGCAGATC

ACAAAGCACGTGGCACAGATCCTGGACTCCCGGATGAACACTAAGTACGA

CGAGAATGACAAGCTGATCCGGGAAGTGAAAGTGATCACCCTGAAGTCC

AAGCTGGTGTCCGATTTCCGGAAGGATTTCCAGTTTTACAAAGTGCGCGA

GATCAACAACTACCACCACGCCCACGACGCCTACCTGAACGCCGTCGTGG

GAACCGCCCTGATCAAAAAGTACCCTAAGCTGGAAAGCGAGTTCGTGTAC

GGCGACTACAAGGTGTACGACGTGCGGAAGATGATCGCCAAGAGCGAGC

AGGAAATCGGCAAGGCTACCGCCAAGTACTTCTTCTACAGCAACATCATG

AACTTTTTCAAGACCGAGATTACCCTGGCCAACGGCGAGATCCGGAAGCG

GCCTCTGATCGAGACAAACGGCGAAACCGGGGAGATCGTGTGGGATAAG

GGCCGGGATTTTGCCACCGTGCGGAAAGTGCTGAGCATGCCCCAAGTGAA

TATCGTGAAAAAGACCGAGGTGCAGACAGGCGGCTTCAGCAAAGAGTCT

ATCCTGCCCAAGAGGAACAGCGATAAGCTGATCGCCAGAAAGAAGGACT

GGGACCCTAAGAAGTACGGCGGCTTCGACAGCCCCACCGTGGCCTATTCT

GTGCTGGTGGTGGCCAAAGTGGAAAAGGGCAAGTCCAAGAAACTGAAGA

GTGTGAAAGAGCTGCTGGGGATCACCATCATGGAAAGAAGCAGCTTCGA

GAAGAATCCCATCGACTTTCTGGAAGCCAAGGGCTACAAAGAAGTGAAA

AAGGACCTGATCATCAAGCTGCCTAAGTACTCCCTGTTCGAGCTGGAAAA

CGGCCGGAAGAGAATGCTGGCCTCTGCCGGCGAACTGCAGAAGGGAAAC

GAACTGGCCCTGCCCTCCAAATATGTGAACTTCCTGTACCTGGCCAGCCAC

TATGAGAAGCTGAAGGGCTCCCCCGAGGATAATGAGCAGAAACAGCTGTT

TGTGGAACAGCACAAGCACTACCTGGACGAGATCATCGAGCAGATCAGC

GAGTTCTCCAAGAGAGTGATCCTGGCCGACGCTAATCTGGACAAAGTGCT

GTCCGCCTACAACAAGCACCGGGATAAGCCCATCAGAGAGCAGGCCGAG

AATATCATCCACCTGTTTACCCTGACCAATCTGGGAGCCCCTGCCGCCTTC

AAGTACTTTGACACCACCATCGACCGGAAGAGGTACACCAGCACCAAAG

AGGTGCTGGACGCCACCCTGATCCACCAGAGCATCACCGGCCTGTACGAG

ACACGGATCGACCTGTCTCAGCTGGGAGGCGACAAAAGGCCGGCGGCCA

CGAAAAAG

Cas9 Sequence in Plasmid#41815 from Addgene.

This is the Cas9 commonly used in our laboratory.

Additionally, the Cas9 sequence in GSE84534 dataset is also this Cas9 sequence.

>Cas9

ATGGACAAGAAGTACTCCATTGGGCTCGATATCGGCACAAACAGCGTC
GGCTGGGCCGTCATTACGGACGAGTACAAGGTGCCGAGCAAAAAATTCAA
AGTTCTGGGCAATACCGATCGCCACAGCATAAAGAAGAACCTCATTGGCGC
CCTCCTGTTCGACTCCGGGGAGACGGCCGAAGCCACGCGGCTCAAAAGAA
CAGCACGGCGCAGATATACCCGCAGAAAGAATCGGATCTGCTACCTGCAGG
AGATCTTTAGTAATGAGATGGCTAAGGTGGATGACTCTTTCTTCCATAGGCT
GGAGGAGTCCTTTTTGGTGGAGGAGGATAAAAAGCACGAGCGCCACCCAA
TCTTTGGCAATATCGTGGACGAGGTGGCGTACCATGAAAAGTACCCAACCA
TATATCATCTGAGGAAGAAGCTTGTAGACAGTACTGATAAGGCTGACTTGC
GGTTGATCTATCTCGCGCTGGCGCATATGATCAAATTTCGGGGACACTTCCT
CATCGAGGGGGACCTGAACCCAGACAACAGCGATGTCGACAAACTCTTTA
TCCAACTGGTTCAGACTTACAATCAGCTTTTCGAAGAGAACCCGATCAACG
CATCCGGAGTTGACGCCAAAGCAATCCTGAGCGCTAGGCTGTCCAAATCCC
GGCGGCTCGAAAACCTCATCGCACAGCTCCCTGGGGAGAAGAAGAACGG
CCTGTTTGGTAATCTTATCGCCCTGTCACTCGGGCTGACCCCCAACTTTAAA
TCTAACTTCGACCTGGCCGAAGATGCCAAGCTTCAACTGAGCAAAGACAC
CTACGATGATGATCTCGACAATCTGCTGGCCCAGATCGGCGACCAGTACGC
AGACCTTTTTTTGGCGGCAAAGAACCTGTCAGACGCCATTCTGCTGAGTGA
TATTCTGCGAGTGAACACGGAGATCACCAAAGCTCCGCTGAGCGCTAGTAT
GATCAAGCGCTATGATGAGCACCACCAAGACTTGACTTTGCTGAAGGCCCT
TGTCAGACAGCAACTGCCTGAGAAGTACAAGGAAATTTTCTTCGATCAGTC

TAAAAATGGCTACGCCGGATACATTGACGGCGGAGCAAGCCAGGAGGAAT
TTTACAAATTTATTAAGCCCATCTTGGAAAAAATGGACGGCACCGAGGAGC
TGCTGGTAAAGCTTAACAGAGAAGATCTGTTGCGCAAACAGCGCACTTTC
GACAATGGAAGCATCCCCCACCAGATTCACCTGGGCGAACTGCACGCTATC
CTCAGGCGGCAAGAGGATTTCTACCCCTTTTTGAAAGATAACAGGGAAAA
GATTGAGAAAATCCTCACATTTCGGATACCCTACTATGTAGGCCCCCTCGCC
CGGGGAAATTCCAGATTCGCGTGGATGACTCGCAAATCAGAAGAGACCAT
CACTCCCTGGAACTTCGAGGAAGTCGTGGATAAGGGGGCCTCTGCCCAGT
CCTTCATCGAAAGGATGACTAACTTTGATAAAAATCTGCCTAACGAAAAGG
TGCTTCCTAAACACTCTCTGCTGTACGAGTACTTCACAGTTTATAACGAGCT
CACCAAGGTCAAATACGTCACAGAAGGGATGAGAAAGCCAGCATTCCTGT
CTGGAGAGCAGAAGAAAGCTATCGTGGACCTCCTCTTCAAGACGAACCGG
AAAGTTACCGTGAAACAGCTCAAAGAAGACTATTTCAAAAAGATTGAATG
TTTCGACTCTGTTGAAATCAGCGGAGTGGAGGATCGCTTCAACGCATCCCT
GGGAACGTATCACGATCTCCTGAAAATCATTAAAGACAAGGACTTCCTGGA
CAATGAGGAGAACGAGGACATTCTTGAGGACATTGTCCTCACCCTTACGTT
GTTTGAAGATAGGGAGATGATTGAAGAACGCTTGAAAACTTACGCTCATCT
CTTCGACGACAAAGTCATGAAACAGCTCAAGAGGCGCCGATATACAGGAT
GGGGGCGGCTGTCAAGAAAACTGATCAATGGGATCCGAGACAAGCAGAGT
GGAAAGACAATCCTGGATTTTCTTAAGTCCGATGGATTTGCCAACCGGAAC
TTCATGCAGTTGATCCATGATGACTCTCTCACCTTTAAGGAGGACATCCAGA
AAGCACAAGTTTCTGGCCAGGGGGACAGTCTTCACGAGCACATCGCTAAT
CTTGCAGGTAGCCCAGCTATCAAAAAGGGAATACTGCAGACCGTTAAGGTC
GTGGATGAACTCGTCAAAGTAATGGGAAGGCATAAGCCCGAGAATATCGTT
ATCGAGATGGCCCGAGAGAACCAAACTACCCAGAAGGGACAGAAGAACA
GTAGGGAAAGGATGAAGAGGATTGAAGAGGGTATAAAAGAACTGGGGTCC
CAAATCCTTAAGGAACACCCAGTTGAAAACACCCAGCTTCAGAATGAGAA
GCTCTACCTGTACTACCTGCAGAACGGCAGGGACATGTACGTGGATCAGGA
ACTGGACATCAATCGGCTCTCCGACTACGACGTGGATCATATCGTGCCCCA
GTCTTTTCTCAAAGATGATTCTATTGATAATAAAGTGTTGACAAGATCCGAT
AAAAATAGAGGGAAGAGTGATAACGTCCCCTCAGAAGAAGTTGTCAAGAA
AATGAAAAATTATTGGCGGCAGCTGCTGAACGCCAAACTGATCACACAAC
GGAAGTTCGATAATCTGACTAAGGCTGAACGAGGTGGCCTGTCTGAGTTGG
ATAAAGCCGGCTTCATCAAAAGGCAGCTTGTTGAGACACGCCAGATCACC
AAGCACGTGGCCCAAATTCTCGATTCACGCATGAACACCAAGTACGATGAA
AATGACAAACTGATTCGAGAGGTGAAAGTTATTACTCTGAAGTCTAAGCTG
GTCTCAGATTTCAGAAAGGACTTTCAGTTTTATAAGGTGAGAGAGATCAAC
AATTACCACCATGCGCATGATGCCTACCTGAATGCAGTGGTAGGCACTGCA
CTTATCAAAAAATATCCCAAGCTTGAATCTGAATTTGTTTACGGAGACTATA
AAGTGTACGATGTTAGGAAAATGATCGCAAAGTCTGAGCAGGAAATAGGC

AAGGCCACCGCTAAGTACTTCTTTTACAGCAATATTATGAATTTTTCAAGA
CCGAGATTACACTGGCCAATGGAGAGATTCGGAAGCGACCACTTATCGAAA
CAAACGGAGAAACAGGAGAAATCGTGTGGGACAAGGGTAGGGATTTCGC
GACAGTCCGGAAGGTCCTGTCCATGCCGCAGGTGAACATCGTTAAAAAGA
CCGAAGTACAGACCGGAGGCTTCTCCAAGGAAAGTATCCTCCCGAAAAGG
AACAGCGACAAGCTGATCGCACGCAAAAAAGATTGGGACCCCAAGAAATA
CGGCGGATTCGATTCTCCTACAGTCGCTTACAGTGTACTGGTTGTGGCCAA
AGTGGAGAAAGGGAAGTCTAAAAAACTCAAAAGCGTCAAGGAACTGCTG
GGCATCACAATCATGGAGCGATCAAGCTTCGAAAAAAACCCCATCGACTTT
CTCGAGGCGAAAGGATATAAAGAGGTCAAAAAAGACCTCATCATTAAGCTT
CCCAAGTACTCTCTCTTTGAGCTTGAAAACGGCCGGAAACGAATGCTCGCT
AGTGCGGGCGAGCTGCAGAAAGGTAACGAGCTGGCACTGCCCTCTAAATA
CGTTAATTTCTTGTATCTGGCCAGCCACTATGAAAAGCTCAAAGGGTCTCCC
GAAGATAATGAGCAGAAGCAGCTGTTCGTGGAACAACACAAACACTACCT
TGATGAGATCATCGAGCAAATAAGCGAATTCTCCAAAAGAGTGATCCTCGC
CGACGCTAACCTCGATAAGGTGCTTTCTGCTTACAATAAGCACAGGGATAA
GCCCATCAGGGAGCAGGCAGAAAACATTATCCACTTGTTTACTCTGACCAA
CTTGGGCGCGCCTGCAGCCTTCAAGTACTTCGACACCACCATAGACAGAA
AGCGGTACACCTCTACAAAGGAGGTCCTGGACGCCACACTGATTCATCAGT
CAATTACGGGGCTCTATGAAACAAGAATCGACCTCTCTCAGCTCGGTGGAG
ACAGCAGGGCTGACCCCAAGAAGAAGAGGAAGGTGTGA

Cas9-Mut Sequence

This is our optimized Cas9 sequence, which has been synonymous mutated based on Plasmid#41815.

>Cas9-Mut

ATGGACAAGAAGTACTCCATTGGGCTCGATATCGGCACAAACAGCGTC
GGCTGGGCCGTCATTACGGACGAGTACAAGGTGCCGAGCAAAAAATTCAA
AGTTCTGGGCAATACCGATCGCCACAGCATAAAGAAGAACCTCATTGGCGC
CCTCCTGTTCGACTCCGGGGAGACGGCCGAAGCCACGCGGCTCAAAAGAA
CAGCACGGCGCAGATATACCCGCAGAAAGAACCGGATCTGTTATTTGCAGG
AGATCTTTAGTAATGAGATGGCTAAGGTGGATGACTCTTTCTTCCATAGGCT
GGAGGAGTCCTTTTTGGTGGAGGAGGATAAAAAGCACGAGCGCCACCCAA
TCTTTGGCAATATCGTGGACGAGGTGGCGTACCATGAAAAGTACCCAACCA

TATATCATCTGAGGAAGAAGCTTGTAGACAGTACTGATAAGGCTGACTTGC
GGTTGATCTATCTCGCGCTGGCGCATATGATCAAATTTCGGGGACACTTCCT
CATCGAGGGGGACCTGAACCCAGACAACAGCGATGTCGACAAACTCTTTA
TCCAACTGGTTCAGACTTACAATCAGCTTTTCGAAGAGAACCCGATCAACG
CATCCGGAGTTGACGCCAAAGCAATCCTGAGCGCTAGGCTGTCCAAATCCC
GGCGGCTCGAAAACCTCATCGCACAGCTCCCTGGGGAGAAGAAGAACGG
CCTGTTTGGTAATCTTATCGCCCTGTCACTCGGGCTGACCCCCAACTTTAAA
TCTAACTTCGACCTGGCCGAAGATGCCAAGCTTCAACTGAGCAAAGACAC
CTACGATGATGATCTCGACAATCTGCTGGCCCAGATCGGCGACCAGTACGC
AGACCTTTTTTTGGCGGCAAAGAACCTGTCAGACGCCATTCTGCTGAGTGA
TATTCTGCGAGTGAACACGGAGATCACCAAAGCTCCGCTGAGCGCTAGTAT
GATCAAGCGCTATGATGAGCACCACCAAGACTTGACTTTGCTGAAGGCCCT
TGTCAGACAGCAACTGCCTGAGAAGTACAAGGAAATTTTCTTCGATCAGTC
TAAAAATGGCTACGCCGGATACATTGACGGCGGAGCAAGCCAGGAGGAAT
TTTACAAATTTATTAAGCCCATCTTGGAAAAAATGGACGGCACCGAGGAGC
TGCTGGTAAAGCTTAACAGAGAAGATCTGTTGCGCAAACAGCGCACTTTC
GACAATGGAAGCATCCCCCACCAGATTCACCTGGGCGAACTGCACGCTATC
CTCAGGCGGCAAGAGGATTTCTACCCCTTTTTGAAAGATAACAGGGAAAA
GATTGAGAAAATCCTCACATTTCGGATACCCTACTATGTAGGCCCCCTCGCC
CGGGGAAATTCCAGATTCGCGTGGATGACTCGCAAATCAGAAGAGACCAT
CACTCCCTGGAACTTCGAGGAAGTCGTGGATAAGGGGGCCTCTGCCCAGT
CCTTCATCGAAAGGATGACTAACTTTGATAAAAATCTGCCTAACGAAAAGG
TGCTTCCTAAACACTCTCTGCTGTACGAGTACTTCACAGTTTATAACGAGCT
CACCAAGGTCAAATACGTCACAGAAGGGATGAGAAAGCCAGCATTCCTGT
CTGGAGAGCAGAAGAAAGCTATCGTGGACCTCCTCTTCAAGACGAACCGG
AAAGTTACCGTGAAACAGCTCAAAGAAGACTATTTCAAAAAGATTGAATG
TTTCGACTCTGTTGAAATCAGCGGAGTGGAGGATCGCTTCAACGCATCCCT
GGGAACGTATCACGATCTCCTGAAAATCATTAAAGACAAGGACTTCCTGGA
CAATGAGGAGAACGAGGACATTCTTGAGGACATTGTCCTCACCCTTACGTT
GTTTGAAGATAGGGAGATGATTGAAGAACGCTTGAAAACTTACGCTCATCT
CTTCGACGACAAAGTCATGAAACAGCTCAAGAGGCGCCGATATACAGGAT
GGGGGCGGCTGTCAAGAAAACTGATCAATGGGATCCGAGACAAGCAGAGT
GGAAAGACAATCCTGGATTTTCTTAAGTCCGATGGATTTGCCAACCGGAAC
TTCATGCAGTTGATCCATGATGACTCTCTCACCTTTAAGGAGGACATCCAGA
AAGCACAAGTTTCTGGCCAGGGGGACAGTCTTCACGAGCACATCGCTAAT
CTTGCAGGTAGCCCAGCTATCAAAAAGGGAATACTGCAGACCGTTAAGGTC
GTGGATGAACTCGTCAAAGTAATGGGAAGGCATAAGCCCGAGAATATCGTT
ATCGAGATGGCCCGAGAGAACCAAACTACCCAGAAGGGACAGAAGAACA
GTAGGGAAAGGATGAAGAGGATTGAAGAGGGTATAAAAGAACTGGGGTCC
CAAATCCTTAAGGAACACCCAGTTGAAAACACCCAGCTTCAGAATGAGAA

GCTCTATCTGTATTATTTGCAGAACGGCAGGGACATGTACGTGGATCAGGA
ACTGGACATCAATCGGCTCTCCGACTACGACGTGGATCATATCGTGCCCCA
GTCTTTTCTCAAAGATGATTCTATTGATAATAAAGTGTTGACAAGATCCGAT
AAAAATAGAGGGAAGAGTGATAACGTCCCCTCAGAAGAAGTTGTCAAGAA
AATGAAAAATTATTGGCGGCAGCTGCTGAACGCCAAACTGATCACACAAC
GGAAGTTCGATAATCTGACTAAGGCTGAACGAGGTGGCCTGTCTGAGTTGG
ATAAAGCCGGCTTCATCAAAAGGCAGCTTGTTGAGACACGCCAGATCACC
AAGCACGTGGCCCAAATTCTCGATTCACGCATGAACACCAAGTACGATGAA
AATGACAAACTGATTCGAGAGGTGAAAGTTATTACTCTGAAGTCTAAGCTG
GTCTCAGATTTCAGAAAGGACTTTCAGTTTTATAAGGTGAGAGAGATCAAC
AATTACCACCATGCGCATGATGCCTACCTGAATGCAGTGGTAGGCACTGCA
CTTATCAAAAAATATCCCAAGCTTGAATCTGAATTTGTTTACGGAGACTATA
AAGTGTACGATGTTAGGAAAATGATCGCAAAGTCTGAGCAGGAAATAGGC
AAGGCCACCGCTAAGTACTTCTTTTACAGCAATATTATGAATTTTTTCAAGA
CCGAGATTACACTGGCCAATGGAGAGATTCGGAAGCGACCACTTATCGAAA
CAAACGGAGAAACAGGAGAAATCGTGTGGGACAAGGGTAGGGATTTCGC
GACAGTCCGGAAGGTCCTGTCCATGCCGCAGGTGAACATCGTTAAAAAGA
CCGAAGTACAGACCGGAGGCTTCTCCAAGGAAAGTATCCTCCCGAAAAGG
AACAGCGACAAGCTGATCGCACGCAAAAAAGATTGGGACCCCAAGAAATA
CGGCGGATTCGATTCTCCTACAGTCGCTTACAGTGTACTGGTTGTGGCCAA
AGTGGAGAAAGGGAAGTCTAAAAAACTCAAAAGCGTCAAGGAACTGCTG
GGCATCACAATCATGGAGCGATCAAGCTTCGAAAAAAACCCCATCGACTTT
CTCGAGGCGAAAGGATATAAAGAGGTCAAAAAAGACCTCATCATTAAGCTT
CCCAAGTACTCTCTCTTTGAGCTTGAAAACGGCCGGAAACGAATGCTCGCT
AGTGCGGGCGAGCTGCAGAAAGGTAACGAGCTGGCACTGCCCTCTAAATA
CGTTAATTTCTTGTATCTGGCCAGCCACTATGAAAAGCTCAAAGGGTCTCCC
GAAGATAATGAGCAGAAGCAGCTGTTCGTGGAACAGCATAAACATTATTTG
GATGAGATCATCGAGCAAATAAGCGAATTCTCCAAAAGAGTGATCCTCGCC
GACGCTAACCTCGATAAGGTGCTTTCTGCTTACAATAAGCACAGGGATAAG
CCCATCAGGGAGCAGGCAGAAAACATTATCCACTTGTTTACTCTGACCAAC
TTGGGCGCGCCTGCAGCCTTCAAGTACTTCGACACCACCATAGACAGAAA
GCGGTACACCTCTACAAAGGAGGTCCTGGACGCCACACTGATTCATCAGTC
AATTACGGGGCTCTATGAAACAAGAATCGACCTCTCTCAGCTCGGTGGAGA
CAGCAGGGCTGACCCCAAGAAGAAGAGGAAGGTGTGA

Reference:

1. Enache, O.M., et al., *Cas9 activates the p53 pathway and selects for p53-inactivating mutations.* Nat Genet, 2020. **52**(7): p. 662-668.
